# The poverty of adult morphology: Bioacoustics, genetics, and internal tadpole morphology reveal a new species of glassfrog (Anura: Centrolenidae: *Ikakogi*) from the Sierra Nevada de Santa Marta, Colombia

**DOI:** 10.1371/journal.pone.0215349

**Published:** 2019-05-08

**Authors:** Marco Rada, Pedro Henrique Dos Santos Dias, José Luis Pérez-Gonzalez, Marvin Anganoy-Criollo, Luis Alberto Rueda-Solano, María Alejandra Pinto-E, Lilia Mejía Quintero, Fernando Vargas-Salinas, Taran Grant

**Affiliations:** 1 Departamento de Zoologia, Instituto de Biociências, Universidade de São Paulo, São Paulo, Brazil; 2 Grupo de Investigación en Biodiversidad y Ecología Aplicada (GIBEA), Facultad de Ciencias Básicas, Universidad del Magdalena, Santa Marta, Colombia; 3 Grupo Herpetológico Universidad del Magdalena, Santa Marta, Colombia; 4 Grupo Biomics, Departmento de Ciencias Biológicas, Universidad de los Andes, Bogotá, Colombia; 5 Grupo de Morfología y Ecología Evolutiva, Universidad Nacional de Colombia, Sede Bogotá, Colombia; 6 Grupo de investigación en Evolución, Ecología y Conservación EECO, Programa de Biología, Facultad de Ciencias Básicas y Tecnologías, Universidad del Quindío, Armenia, Colombia; Universitat Trier, GERMANY

## Abstract

*Ikakogi* is a behaviorally and morphologically intriguing genus of glassfrog. Using tadpole morphology, vocalizations, and DNA, a new species is described from the Sierra Nevada de Santa Marta (SNSM), an isolated mountain range in northern Colombia. The new taxon is the second known species of the genus *Ikakogi* and is morphologically identical to *I*. *tayrona* (except for some larval characters) but differs by its genetic distance (14.8% in mitochondrial encoded cytochrome *b MT-CYB*; ca. 371 bp) and by the dominant frequency of its advertisement call (2928–3273 Hz in contrast to 2650–2870 Hz in *I*. *tayrona*). They also differ in the number of lateral buccal floor papillae, and the position of the buccal roof arena papillae. Additionally, the new species is differentiated from all other species of Centrolenidae by the following traits: tympanum visible, vomerine teeth absent, humeral spines present in adult males, bones in life white with pale green in epiphyses, minute punctuations present on green skin dorsum, and flanks with lateral row of small, enameled dots that extend from below eye to just posterior to arm insertion. We describe the external and internal larval morphology of the new species and we redescribe the larval morphology of *Ikakogi tayrona* on the basis of field collected specimens representing several stages of development from early to late metamorphosis. We discuss the relevance of larval morphology for the taxonomy and systematics of *Ikakogi* and other centrolenid genera. Finally, we document intraspecific larval variation in meristic characters and ontogenetic changes in eye size, coloration, and labial tooth-rows formulas, and compare tadpoles of related species. *Ikakogi tayrona* has been proposed as the sister taxon of all other Centrolenidae; our observations and new species description offers insights about the ancestral character-states of adults, egg clutches, and larval features in this lineage of frogs.

## Introduction

Glassfrogs, family Centrolenidae [1], are a charismatic group comprising 158 species restricted to the Neotropics from southern Mexico to northern Argentina [2]. They are renowned for their partially or totally transparent ventral skin, reproductive behavior closely associated with streams, and by the elaborated parental care performed by adult males [3–5] or females [6].

The last decade witnessed a great increase in the knowledge of phylogenetics (e.g. [5, 7–11]), taxonomy (e.g. [4, 5, 12–16]), and biology/ecology of glassfrogs (e.g. [17, 18, 6]). However, the alpha diversity within the family remains underestimated. As more remote, unexplored areas are surveyed, more species are discovered, as the results of recent studies in Ecuador (e.g. [19, 20]), Peru [21, 22, 11] and Brazil [23, 14] have demonstrated. It is expected that Colombia will follow the same pattern of hidden diversity.

Currently, 78 species of centrolenids have been reported from Colombia, representing more than 50% of the total diversity of the family [24]. Many areas remain to be properly explored, such as the Sierra Nevada de Santa Marta (SNSM), home of the monotypic genus *Ikakogi* [5]. The genus *Ikakogi* is particularly interesting for the systematics of centrolenids due to the uncertainty of its placement. Some authors (i.e. [5]) concluded that it should be left as *incerta sedis* within Centrolenidae, whereas others [10] suggested that it could be the sister taxa of Hyalinobatrachiinae + Centroleninae (see also [7]). The genus is also of great interest for biogeographical studies, given its endemic distribution in the long isolated massif of the SNSM.

Nevertheless, *Ikakogi tayrona* is still a poorly known taxon. Recently, during fieldwork on the northern flank of SNSM in Colombia, we collected several specimens of a glassfrog allied to *I*. *tayrona* that we hypothesized could be a different species. Here we provide evidence from genetic andbioacoustic analyses as well as tadpole morphology to support our hypothesis of a new species. In addition, we redescribe the fossorial tadpoles of *I*. *tayrona*, discuss the larval specialization for burrowing and the relevance of internal larval morphology (i.e. buccopharingeal anatomy, cranial muscles, skeleton) for the taxonomy and systematics of *Ikakogi* and centrolenid genera.

## Materials and methods

### Adult morphology

Species detection was carried out by using visual encounter surveys (VES). Specimens were collected by hand, euthanized by topical application of 20% benzocaine anesthetic mixed with water, fixed in 10% formalin after collection of tissue samples for DNA analysis (preserved in 96% ethanol), and preserved in 70% ethanol. Although euthanized methods were not evaluated by an institutional animal care and use committee or similar regional ethics committee, voucher collections strictly complied with the ethical conditions as dictated by the environmental authorities of Colombia (see scientific license numbers below). Collection of amphibians involve *Ikakogi tayrona*, a species recognized as vulnerable (VU) by the IUCN red list category. Field collection in Magdalena department was authorized by the Ministerio de Ambiente, Vivienda y Desarrollo Territorial de Colombia, Corporación Autónoma Regional del Magdalena (Resolución 0425 de 2015) and in Guajira department by the Corporación Autónoma Regional de la Guajira, proyecto Evaluación Ecológica de las Ranas Arlequines Habitantes Cuencas Altas Rio Palomino, Ancho y Jerez (Contrato 0008 de 2009). Measurements of preserved and live specimens adults were based on [25, 26, 4] and were taken using a digital Mitutoyo caliper to the nearest 0.1 mm. Unless otherwise noted, measurements and proportions are reported only for adults, as determined by examination of gonads and presence of secondary sexual characteristics (i.e., vocal slits, nuptial pads and humeral spine in males). Summaries of measurements are reported as the mean, plus ± standard deviation (SD). Descriptive terminology and definitions follow those of [27, 28, 4]. For ease of comparison, diagnostic characters are in agreement with the proposals of [3, 25, 4]. Webbing formulae follow the convention proposed by [29] with modifications by [30]. Fingers are numbered preaxially to postaxially from I–IV; although this disagrees with the hypothesis that digit I was lost in anurans [31, 32] traditionally authors have used this numbering convention in centrolenid taxonomy. Ecological and reproductive data and color in life were derived from photos and field notes by MAR deposited at Instituto de Ciencias Naturales, Universidad Nacional de Colombia, Colombia (ICN). Generic allocation follows [5]. We examined and described external vocal sac structure and employed the terminology of [33–35].

Voucher specimens are deposited in the ICN and Colección herpetológica de la Universidad del Magdalena, Santa Marta—Colombia (CBUMAG: ANF). The abbreviations for field series numbers used throughout the text are: MAR = Marco Antonio Rada and RC = José Rances Caicedo.

### Call description and bioacoustic analysis

The description of the advertisement call is based on 17 calls recorded from five males of Ikakogi sp. Body size (snout–vent length, SVL) of two of the calling males was measured using a digital Mitutoyo caliper (model type 500-196-20, accuracy±0.1 mm). Voucher specimens collected in this study and SVL for each specimen are reported in S2 Appendix. Calls were recorded at night using a Marantz PMD660 recorder coupled to a Sennheiser ME-66 microphone at a distance of 0.5–1.0 m from the calling frog. The recording level was adjusted manually during each session to obtain the best signal-to-noise ratio and to avoid distortion. Sounds were recorded using sampling rates of 44.1 Hz and a resolution of 24 bits and saved in an uncompressed wav (wave) format. Frog body temperature was measured at the time of recording using an Extech infrared thermometer (accuracy 0.1°C). Advertisement calls were analyzed using the software Raven Pro v. 1.4 for Windows. Temporal properties were obtained from oscillograms (temporal resolution = 5.33 ms) and spectral information was obtained from a power spectrum elaborated using Fast Fourier Transformation (points Blackman window, 256 points). The terminology and procedures for measuring spectral and temporal call traits followed [36]. Descriptions of temporal and spectral structure of calls for all species include the following variables: (1) call duration (s), (2) number of pulses per call, (3) pulse duration (s), (4) dominant frequency averaged across all notes of a call (Hz), and (5) low and high frequency measured at 20 dB (re 20 mPA) below the peak intensity of the dominant frequency (value at which the signal could still be clearly distinguished from the background noise). Our sampling unit for statistical analysis was the recorded individual. Recordings are deposited at Fonoteca Zoológica, Museo Nacional de Ciencias Naturales, CSIC, Madrid, Spain (http://www.fonozoo.com/; Codes FZ Sound Collection 10045–48).

### Tadpole description

In April 2015 and October 2016, we collected 60 tadpoles of *Ikakogi* tayrona and 15 of the undescribed species (S1 Appendix). These larvae were euthanized by immersion in 1% ethanol + benzocaine anesthetic and preserved in 5% formalin. Development stages were delimited following [37]. Morphological measurements and terminology for larval characters were noted following [38, 39]. Comparative data of related species were taken from tadpole descriptions available in the literature [34–55]. General descriptions were based on individuals in stage 35 as recommended by [49], but we also measured another 15 tadpoles between stages 25–35 of the undescribed species (CBUMAG: ANF 01015–16) and 26 tadpoles between stages 24–39 of *I*. *tayrona* (CBUMAG: ANF 00960, 01017 and ICN 58308). A digital Vernier caliper (model type IP54, accuracy±0.01 mm) in a Zeiss dissecting stereomicroscope was used for measurements in *I*. *tayrona* (86 individuals), whereas a Mitutoyo series 500 digital caliper (model type CD-8" CSX accuracy±0.01 mm) was used for specimens of the new species (15 individuals).

### Larval muscles (cranial and axial), skeleton (chondrocranium and hyobranchial apparatus) and buccopharyngeal morphology

Two tadpoles of each species were dissected according to [56] in order to visualize the buccopharyngeal characters (stages 27, 29 for *Ikakogi tayrona*, ICN 58308 and 28, 35 for *Ikakogi* sp., CBUMAG: ANF 01015). One individual per species (stages 29 for *Ikakogi tayrona* and 28 for *Ikakogi* sp., CBUMAG: ANF 00960 and CBUMAG: ANF 01015 respectively) were submitted to the protocol of [57] for scanning electron microscopy (SEM). For the study of musculoskeletal system (chondocranium, hiobranquial apparatus and cranial muscles), three additional tadpoles (stages 35 and 39 for *Ikakogi tayrona* and 28 for *Ikakogi* sp., ICN 58310 and CBUMAG: ANF 01018, and CBUMAG: ANF 01016, respectively) were cleared and stained according to [58] with modifications as described by [59]. This procedure was interrupted after staining with Alcian blue and the muscles exposed by dissection. Following inspection of muscle characters, we continued with the clearing process to visualize the chondrocranium. Terminology follows [60–63] for musculo-skeletal characters and [56, 64] for buccopharyngeal structures.

### Egg clutches and oviposition site features

Reproductive behavior was observed at two localities at Sierra Nevada de Santa Marta, SNMS; see S1 Appendix. Between April 1–2, 2015, and October 1, 2016 we performed visual encounter surveys alongside streams. For each egg clutch of *Ikakogi tayrona* and *Ikakogi* sp., we noted the substrate (e.g., leaves, stems), position on the substrate (e.g., upper or lower side of leaves), and height above the ground or water. The clutches were attributed to the species because a female was guarding eggs. For each egg clutch observed we noted number and size (diameter) of eggs, development stage and color of embryos. Quantitative features of oviposition site and egg clutches are reported as mean ± SD (range).

### Genetic distances

DNA was extracted from ethanol-preserved thigh muscle, with the DNeasy (QIAGEN, Valencia, CA) isolation kit. Amplification of cytochrome *b MT-CYB* sequence, ca. 371 bp, was carried out in a 25μl reaction using the primers of [65, 66] and a PCR Master Mix (2X) (Thermo Fisher Scientific Inc., USA). For DNA amplifications, the PCR program included an initial denaturing step of 30s at 96°C, followed by 35 cycles of amplification (96°C for 30 s; 48–54°C for 30 s; 60°C for 60s), with a final extension step at 60°C for 7 min. PCR amplification products were cleaned using the Agencourt AMPure XP DNA Purification and Cleanup kit (Beckman Coulter Genomics, Brea, CA, USA), and were sequenced in both directions to check for potential errors by a third party using fluorescent dye labeled terminators (ABI Prism Big Dye Terminators v. 1.1 cycle sequencing kits; Applied Biosystems, USA) with an ABI 3730XL DNA Analyzer (Applied Biosystems, USA). Chromatograms obtained were read and contigs made using the sequence editing software Sequencher v5.3 [67]. Sequence variation of the static alignment of mitochondrial Cytochrome b2 (Cytb2) gene were used to calculate the uncorrected pairwise genetic distances in Sequencher v5.3 [67], S3 Appendix. The generated sequences were deposited in GenBank under accession numbers MK809522–MK809523, S4 Appendix.

### Nomenclatural acts

The electronic edition of this article conforms to the requirements of the amended International Code of Zoological Nomenclature, and hence the new names contained herein are available under that Code from the electronic edition of this article. This published work and the nomenclatural acts it contains have been registered in ZooBank, the online registration system for the ICZN. The ZooBank LSIDs (Life Science Identifiers) can be resolved and the associated information viewed through any standard web browser by appending the LSID to the prefix “http://zoobank.org/”. The LSID for this publication is urn:lsid:zoobank.org:pub:73CD6D36-B4DB-4120-9A43-69DD0EEE0668. The electronic edition of this work was published in a journal with an ISSN, and has been archived and is available from the following digital repositories: PubMed Central, LOCKSS.

## Results

### Description of the new species

**Fig 1:**
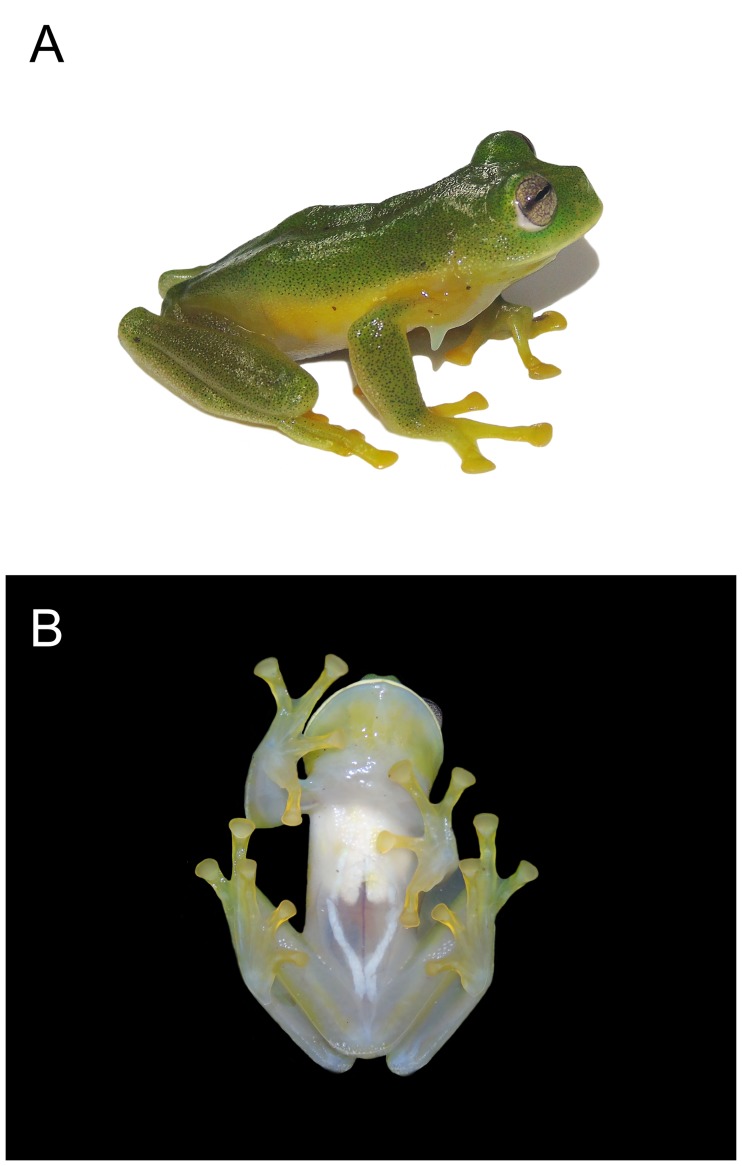
*Ikakogi ispacue* sp. nov. (A) Dorsal and (B) ventral view of holotype (SVL = 29.6 mm; ICN 56204; male; photos not to scale).

**Fig 2:**
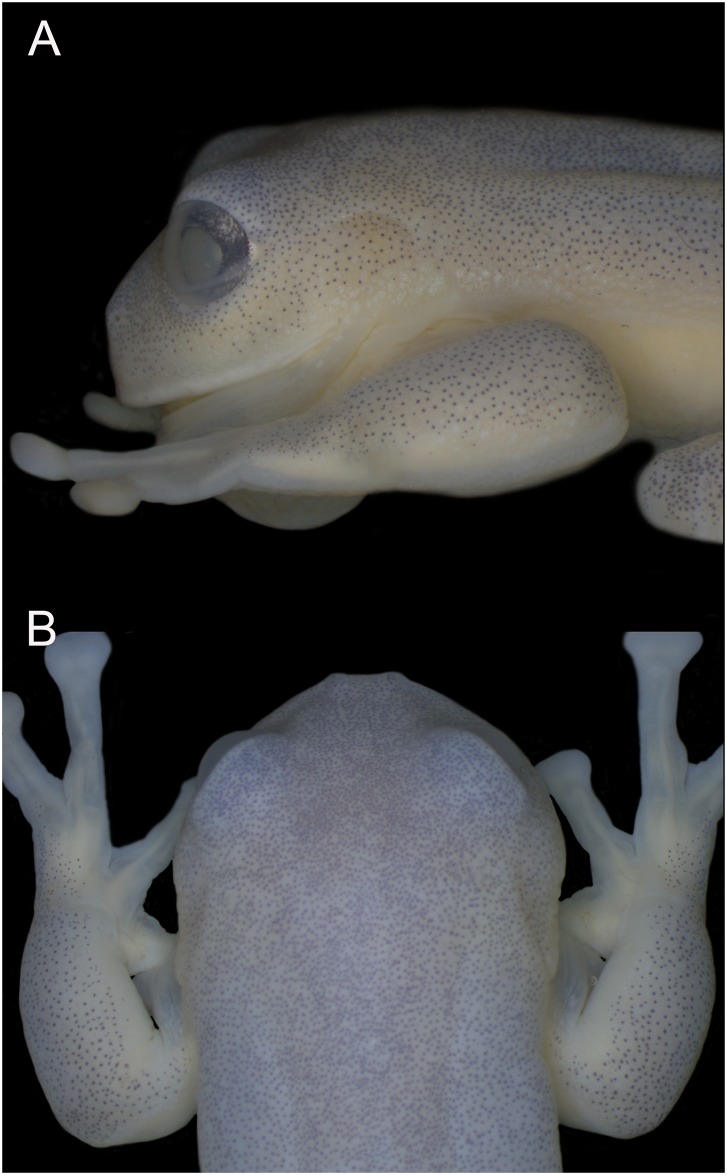
Snout shape of *Ikakogi ispacue* sp. nov. Lateral (A) and dorsal (B) snout of *Ikakogi ispacue* sp. nov., holotype (ICN 56204; male).

**Fig 3:**
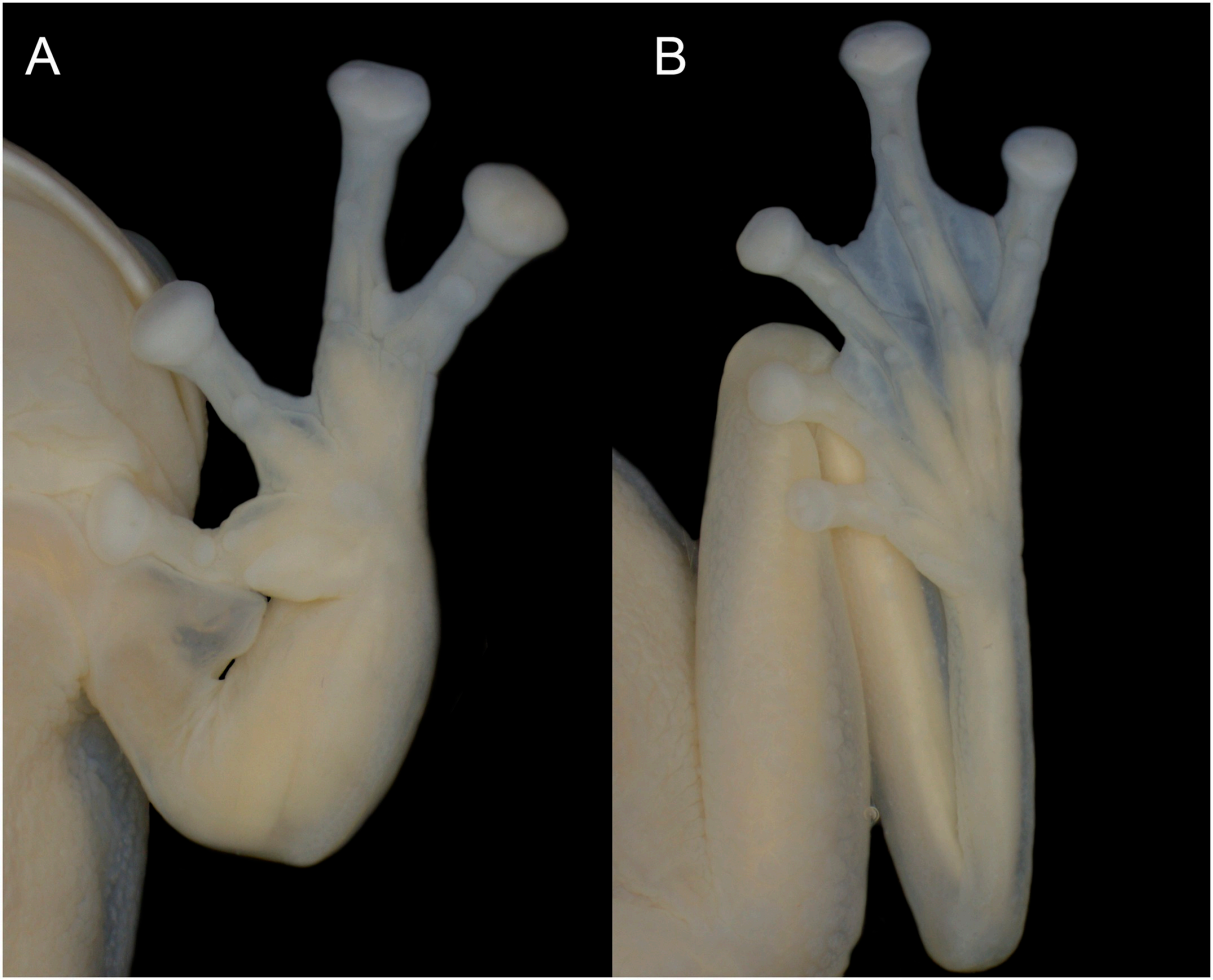
*Ikakogi ispacue* sp. nov. holotype (ICN 56204; male). (A) hand and (B) foot. Scale bar equal to 2 mm.

**Fig 4:**
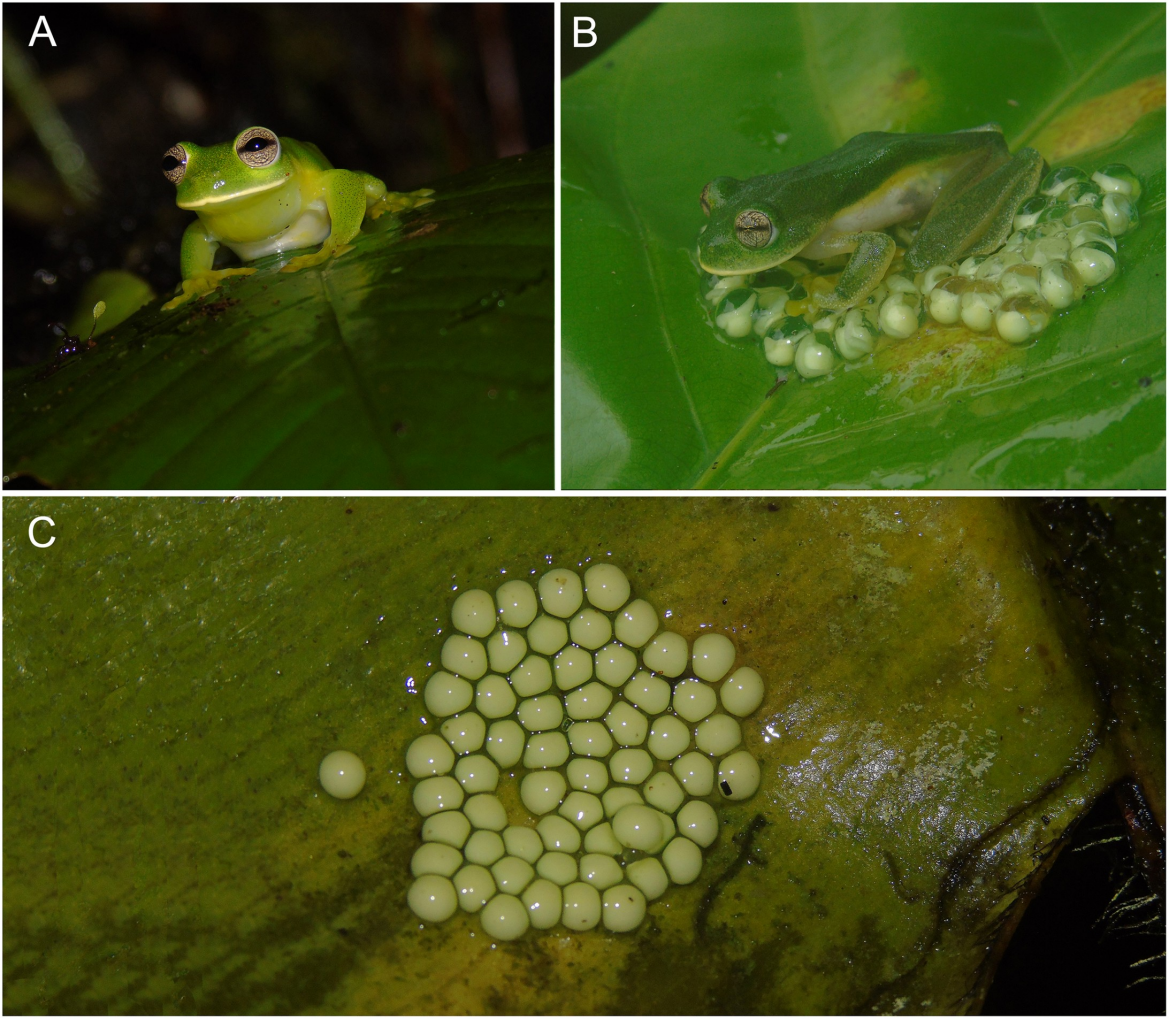
*Ikakogi ispacue* sp. nov. Adult male (A), holotype (ICN 56204; SVL 29.6 mm), adult female (B), paratype (ICN 56202; SVL 29.5 mm) and egg mass (C) of *Ikakogi ispacue* sp. nov. Note the empty space in the middle of the array and unpigmented eggs in a clear jelly. Photos not to scale.

*Ikakogi ispacue* sp. nov. (Figs 1–4), urn:lsid:zoobank.org:act:7580348C-1F56-4286-8DDD 21CD45ADCE78.

#### Holotype

ICN 56204 (MAR3626) an adult male collected by Marco Rada, Luis Alberto Rueda-Solano and Jose Luis Pérez-González on 1 October, 2016.

#### Type locality

Small tributary of the Palomino River (11,058091/ -73.227770; 950 m asl; WGS 84 origin), N flank of Sierra Nevada de Santa Marta, Corregimiento of Palomino, Wimangaga locality, Dibulla Municipality, Guajira Department, Colombia.

#### Paratypes

CBUMAG: ANF 00938 (LARS 112) and ICN 56198 (LARS 111)–ICN 56199 (LARS 201), ICN 56200 (JOC109), adult males, same locality as the holotype, collected on April 1, 2015 by Fernando Vargas-Salinas, Jose Luis Pérez-González and Luis Alberto Rueda-Solano; ICN 56201, 56203, 56205–09 (MAR3623, 3625, 3627–31), adult males, same data as the holotype, collected on October 1, 2016 by Marco Rada, Luis Alberto Rueda-Solano and Jose Luis Pérez-González; ICN 56202 (MAR3624), ICN 56210 (MAR3632), adult females, same data and locality as the holotype; ICN 58309; CBUMAG: ANF 01015–16, tadpoles, same locality as the holotype.

#### Etymology

The specific epithet originates from the Kogi words “*tshi* and *“spákue*”, meaning “twin of”. The word is used as noun in apposition and refers to the high similarity and presumed close relationships of the new species and *Ikakogi tayrona*.

#### Generic placement

The new species is referred to as *Ikakogi* on the basis of phenotypic characters observed in ‬the genus *sensu* [5]: i) presence of humeral spines in adult males (Fig 1A), and an extremely large *crista medialis* which extends along the entire length of the humerus; ii) presence of white bones in life (but see below); iii) a ventral parietal peritoneum white anterior and transparent posterior (Fig 1B); iv) a hepatic and visceral peritonea transparent, and, v) females that guard clutches (Fig 2B).

#### Definition

(1) vomerine teeth absent; (2) snout rounded in dorsal profile and slightly sloping or truncate in lateral profile (Fig 2); (3) tympanum not visible (tympanic membrane and annulus not differentiated; supratympanic fold present (Figs 1A and 2A); (4) dorsal skin smooth (5) skin on belly and ventral thighs granular; pair of tubercles on ventral surfaces of thighs below vent large, round, flat; subcloacal skin granular, not enameled; (6) parietal peritoneum covered by iridophores, 1/2 of the ventral surface of the belly; visceral peritoneum translucent (Fig 1B); (7) liver tetra-lobed; (8) humeral spines in adult males present (*crista ventralis* protruding (Figs 1A and 3A); (9) webbing between Fingers I–III basal: I (2^+^ − (2^1/2^ − 2^+^)), II (1^1/2^ − 3^1/2^) − (2^+^ − 3^+^) (Fig 3A); webbing of outer fingers: III (2 − 2^−^) − (2^+^ − 2^+^) IV; (10) webbing between toes: I (1 − (2 − 2^+^) II (1^−^ − 2) − (1 − 2^1/4^) III (1 − 2) − (1^−^ − 2^1/2^) IV (2^+^ − 1^−^) − (2^1/2^ − 1^1/2^) V (Fig 3B); (11) ulnar fold present, low; tarsal fold present, low (12) nuptial excrescence unpigmented, Type I, prepollex distinct, forming a elongate “prepollical bulge” at the base of the thumb but not piercing the skin (Fig 1B); (13) when adpressed, Finger I slightly larger than Finger II; (14) disc of Finger III width about 60–64% of eye diameter; (15) color in life, dorsum green with minute black punctuations (a smallest mark formed by just one star-like melanophore) along dorsal surfaces and a lateral row of small enameled dots that extends from below the eye to just posterior to the insertion of the arm (Figs 1A and 2A); color of bones white (diaphysis) but with accumulation of biliverdin in bone ephiphysis (very pale green coloration) (Fig 1A and 1B); (16) color in preservative, dorsum very pale lavender to cream with punctuations (melanophores) along dorsal surfaces (Fig 2A and 2B); (17) iris coloration in life, golden to copper with fine dark brown reticulations and dark colored area toward the midline; (18) dorsal surfaces of fingers and toes I-IV lacking melanophores; (19) males call from the upper and lower surfaces of leaves (Fig 2A); the call is a single high-pitched note (“trill”) with multiple pulses and a mean call duration of 0.122 ± 0.041 s (0.082–0.185 s); dominant frequency at 3129 ± 147 Hz (2928–3273 Hz); (21) eggs pale cream or yellowish, deposited on the underside of leaves over streams (n = 3; Fig 2A and 2B), clutches containing uniformly pale cream or pale green eggs (n = 4; 55 ± 6.21; 48–62 eggs; females attend egg clutches located either on the upper or underside of leaves overhanging streams; (22) reddish tadpole; snout acuminated in dorsal and lateral views; (23) upper jaw sheath with conspicuous serrated edge and arch-shape; (24) labial tooth row formula 2(2)/3; (25) 13 small lateral buccal floor papillae (n = 2); (26) five conical papillae present in the buccal roof arena, three of them are lateral and two are medial located (n = 2); (27) basihyal (copula I) absent (but see Discussion section); (28) the anterior margin of trabercular horns is rounded without medial projection; (29) the oculomotor foramen is closed totally; and (30) snout–vent length (SVL) in adult males 28.7–30.2 mm (n = 9; 29.4 ± 0.4), and adult females 29.5–30.6 mm (n = 2; 30.1 ± 0.8; Table 1).

**Table 1:** Measurements (mm) of adult *Ikakogi ispacue* sp. nov. See text for abbreviations.

latMeasurement	Males (*n* = 9)				Females (*n* = 2)			
	min	max	mean	SD	min	max	mean	SD
SVL	28.7	30.2	29.4	0.4	29.5	30.6	30.1	0.8
TL	16.2	17.0	16.6	0.3	16.4	16.9	16.6	0.3
FL	13.0	13.9	13.4	0.4	13.3	13.7	13.5	0.3
HL	8.3	8.8	8.6	0.2	8.5	8.8	8.6	0.3
HW	10.8	11.3	11.1	0.2	11.1	11.6	11.4	0.4
IOD	4.5	4.8	4.6	0.1	4.8	5.0	4.9	1.0
IN	1.7	2.3	2.0	0.2	1.7	2.0	1.9	0.2
ED	3.2	3.6	3.4	0.1	3.4	3.5	3.5	0.1
EN	2.3	2.4	2.3	0.04	2.4	2.7	2.5	0.3
IN	1.7	2.3	2.0	0.2	1.7	2.0	1.9	0.2
3WD	2.0	2.3	2.2	0.1	2.1	2.1	2.1	0.01

#### Diagnosis

*Ikakogi ispacue* sp. nov. can be distinguished from other centrolenids (the only exception is *I*. *tayrona*) by having a slightly sloping snout, rounded lateral profile; a tympanum that is not visible; humeral spines in adult males; vomerine teeth absent; parietal peritoneum 1/2 white; green dorsum with black punctuations along the dorsal surfaces and a flanks with a lateral row of small enameled dots that extends from below the eye to just posterior to the insertion of the arm; color of bones in life white, but with a pale green coloration in bone epiphysis; color in preservative uniformly very pale lavender to cream.

Flanks with a lateral row of small enameled dots and white bones are unusual in centrolenids and are otherwise known to occur only in some species of *Centrolene* [68, 69], in *Ikakogi tayrona* (see [70]), in *Nymphargus* [4] and *Hyalinobatrachium* [71]. Species of *Centrolene* having white dots in an area that extends from below the eye to the insertion of the arm include *C*. *antioquiense* [72]; *C*. *bacatum* [68]; *C*. *buckleyi* [73]; *C*. *daidaleum* [74]; *C*. *heloderma* [75], *C*. *huilense* [69], *C*. *pipilatum* [27]; *C*. *peristictum* [27]; *C*. *sabini* [76], *C*. *savagei* [74]; *C*. *scirtetes* [77]; *C*. *solitaria* [74], *C*. *venezuelense* [78] and *C*. *robledoi* [69]. However, *I*. *ispacue* sp. nov. has a tympanum that is not visible (tympanic membrane and annulus not differentiated) whereas all the species mentioned above have a tympanum that is visible and the tympanic membrane and annulus are clearly differentiated (the exceptions are *Centrolene buckleyi* and *C*. *venezuelense* in which some populations have a tympanum concealed and neither the membrane nor the annulus is evident; (see [4, 7]). In addition, preserved *I*. *ispacue* sp. nov. have a uniform dorsal coloration, with very pale lavender to cream punctuations (lavender with distinctive marks, clear and/or dark spots). Regarding the color bones in life, of the 35 known species of *Nymphargus*, only two have white bones: *N*. *anomalus* [27] and *N*. *rosada* [79]. *Ikakogi ispacue* sp. nov., is easily distinguished from those taxa by having white bones with pale green coloration in bone epiphyses and the adult males having humeral spines (humeral spine absent in adult males of *N*. *anomalus* and *N*. *rosada*). Further, the color in life in *I*. *ispacue* sp. nov. is uniform green with black punctuations, whereas the dorsum in *N*. *anomalus* is tan-brown to very pale green olive with black ocelli surrounding orange spots. Moreover, the dorsum in *N*. *rosada* is tan-brown with orange dots [27, 79]. Species of *Hyalinobatrachium* are yellowish green or lime green and have distinctive marks like flecks, dots, and spots on their dorsal skin (see [4]). However, in *I*. *ispacue* sp. nov. the dorsum is uniformly green and only has minute star-like punctuations (Fig 3B).

**Fig 5:**
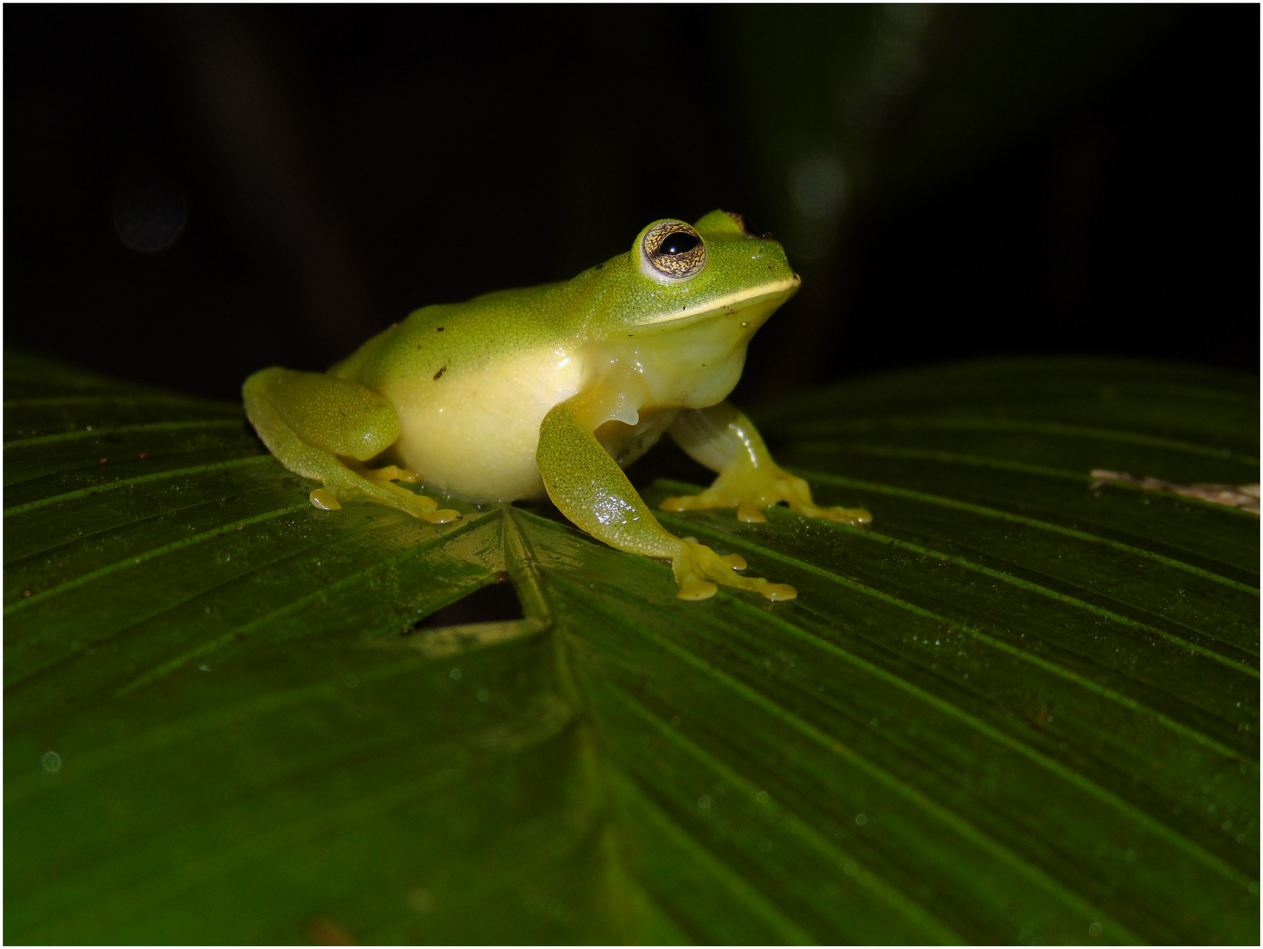
Adult male of *Ikakogi tayrona* from type locality. (SVL = 30.5 mm; not collected).

**Fig 6:**
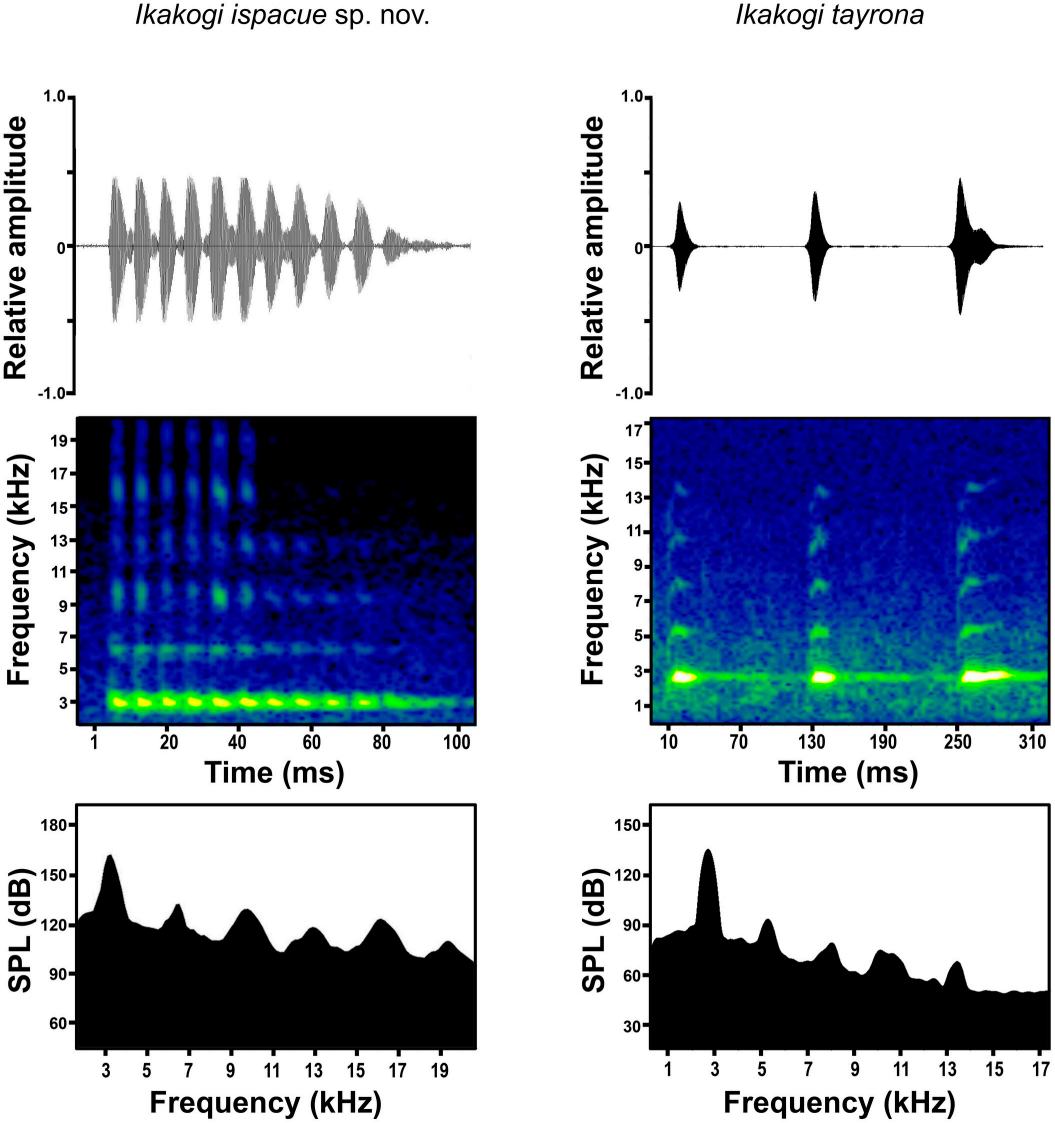
Oscillogram, audiospectrogram and power spectrum of advertisement calls of *Ikakogi ispacue* sp. nov. (paratype, CBUMAG: ANF 00938, SVL 33.5 mm, 19.9 °C) and *Ikakogi tayrona*. Note the difference in the temporal features of the call between species.

The adults of *Ikakogi ispacue* sp. nov. are morphologically identical to *I*. *tayrona* (Fig 5), but differ in their cytochrome B sequence and their advertisement call (Fig 6A and 6B). Genetic distances between both species reveal a noteworthy diversity, uncorrected *MT-Cytb2* pairwise distance of 14.8% (identical sites 316 bp = 85.2%; 55 differences; S3 Appendix). This values strongly indicates that the two populations are not conspecific. On the other hand, the call of *I*. *ispacue* sp. nov. is a single high-pitched note (“trill”) with multiple pulses and a high dominant frequency (2928–3273 Hz); whereas the call of *I*. *tayrona* is described as 3–4 well-defined non-pulsed, “beep” notes with a dominant frequency of 2650–2870 Hz ([80], Table 2). Additionally, the tadpole of *I*. *ispacue* sp. nov. is almost indistinguishable from the larvae of *I*. *tayrona* externally. However they differ in their internal morphology; specifically, they differ in the number of lateral buccal floor papillae (13 in *I*. *ispacue* sp. nov., n = 2, in contrast to 10 in *I*. *tayrona*, n = 2) and the position of the buccal roof arena papillae (five papillae distributed laterally in *I*. *tayrona*; three papillae latterally and two papillae medially in *I*. *ispacue* sp. nov. Additionally, the basihyal was absent in the larvae of *Ikakogi ispacue* sp. nov., n = 2 but present in *I*. *tayrona* (n = 2; but see Discussion, below).

**Table 2:** Summarized features of the advertisement call of *Ikakogi ispacue* sp. nov. and *I*. *tayrona*. * Indicate data obtained from literature.

Species	SVL (mm)	Temperature (°C)	Number of calls recorded	Call duration (ms)	Number of notes	Number of pulses	Call structure	Dominant Frequency (Hz)
*I*. *ispacue* sp. nov.	32.7–33.5	19.9–20.6	17	122 ± 41(82–185)	1	11–20	Pulsed	3129 ± 146.5 (2928–3273)
*I*. tayrona*	31.7–35.0	14.4–16.2	85	269 ± 31 (203–316)	3–4	Absent	Tonal	2710 ± 100.0 (2650–2870)

#### Description of holotype

A moderate-sized glassfrog, SVL in adult male 29.6 mm (adult male); head slightly wider than body; HW 38% of SLV; head wider than long, HW/HL = 1.3 snout slightly sloping in dorsal profile and truncated in lateral profile; loreal region concave, nostrils elevated, delimiting a slight depression in the internarial area; canthus rostralis round; lips slightly flared; eyes large, directed anterolaterally at ca., 45°; ED equals 31% of HW; supratympanic fold distinct, tympanic annulus undistinguished; tympanic membrane not differentiated, IN 55% of ED; choanae medium sized, ovoid, separated; vomerine teeth absent; tongue round, with posterior distal margin slightly notched, posterior edge not adherent to floor of mouth; vocal slits elongated, extending posterolaterally from base of tongue to the angle of jaws; large, external, subgular vocal sac.

Humeral spine present; forearm robust; ulnar folds present, low; Finger I slightly longer than Finger II, discs subtruncate; prepollex distinct, forming a elongate “prepollical bulge” at the base of the thumb but not piercing the skin; webbing formula III (2 − 2^−^) − (2^+^ − 2^+^) IV; subarticular tubercles round, supernumerary tubercles at base of fingers, palmar tubercle slightly longer than wide, thenar tubercle elliptical, longer than palmar tubercle; nuptial excrescences Type I. Hind limbs slender, TL 57.3% of SLV; tarsal fold present, low, tarsal tubercles absent; inner metatarsal tubercle elliptic, outer metatarsal tubercle absent; subarticular tubercles round; formulae on feet I (1 − (2 − 2^+^) II (1^−^ − 2) − (1 − 2 ^1/4^) III (1 − 2) − (1^−^ − 2^1/2^) IV (2^+^ − 1^−^) − (2^1/2^ − 1^1/2^) V; toe discs equal to finger discs. Skin of dorsal surfaces smooth; venter skin on belly and thighs granular; cloacal opening directed posteriorly; pair of enlarged flat warts on posteroventral surface of thighs; subcloacal skin granular and enameled.

#### Color in life

Based on color photographs and MAR´s field notes of holotype and paratypes ICN (56204, 56198–99; 56200–01; 56203–09) and CBUMAG:ANF (00938). Dorsum green with minute black punctuations; margin of upper lip, flanks creamy yellowish; flanks with a very minute lateral row of enameled (white) dots; fingers and toes yellow; ventral surface, including limbs cream; parietal peritoneum white (iridophores) covering 1/2 section of the abdominal region; heart white, not visible; visceral peritonea translucent; iris coloration golden with fine dark brown reticulations and dark colored area toward the midline; bones manly white (diaphysis) but with a very pale green coloration in bone ephiphysis (presumably by accumulation of biliverdin).

#### Color in preservative (ethanol 70%)

Dorsum of head, body and limbs cream to very pale lavender covered with small melanophores (except finger and toes I–IV). Throat, chest, hands, feet, and lower surfaces of legs cream; flanks with a very minute lateral row of white dots (Fig 2A); venter cream; iris silvery with dark reticulation (Fig 2A).

#### Measurements of holotype (in mm)

SVL = 29.6; tibia length = 17.0; foot length = 13.7; head length = 8.3; head width = 11.2; horizontal eye diameter = 3.5; interorbital distance = 4.5; eye—nostril distance = 2.4; internarial distance = 1.9; width of disc on the finger III = 2.0.

#### Variation

Females are about 2.3% larger than males. Measurements of the type series are summarized in Table 1. No other morphological differences are observed between males and females, except for the sexually dimorphic structures (i.e., nuptial pads and vocal slits in males and convoluted oviducts in females).

#### Geographic distribution

**Fig 7:**
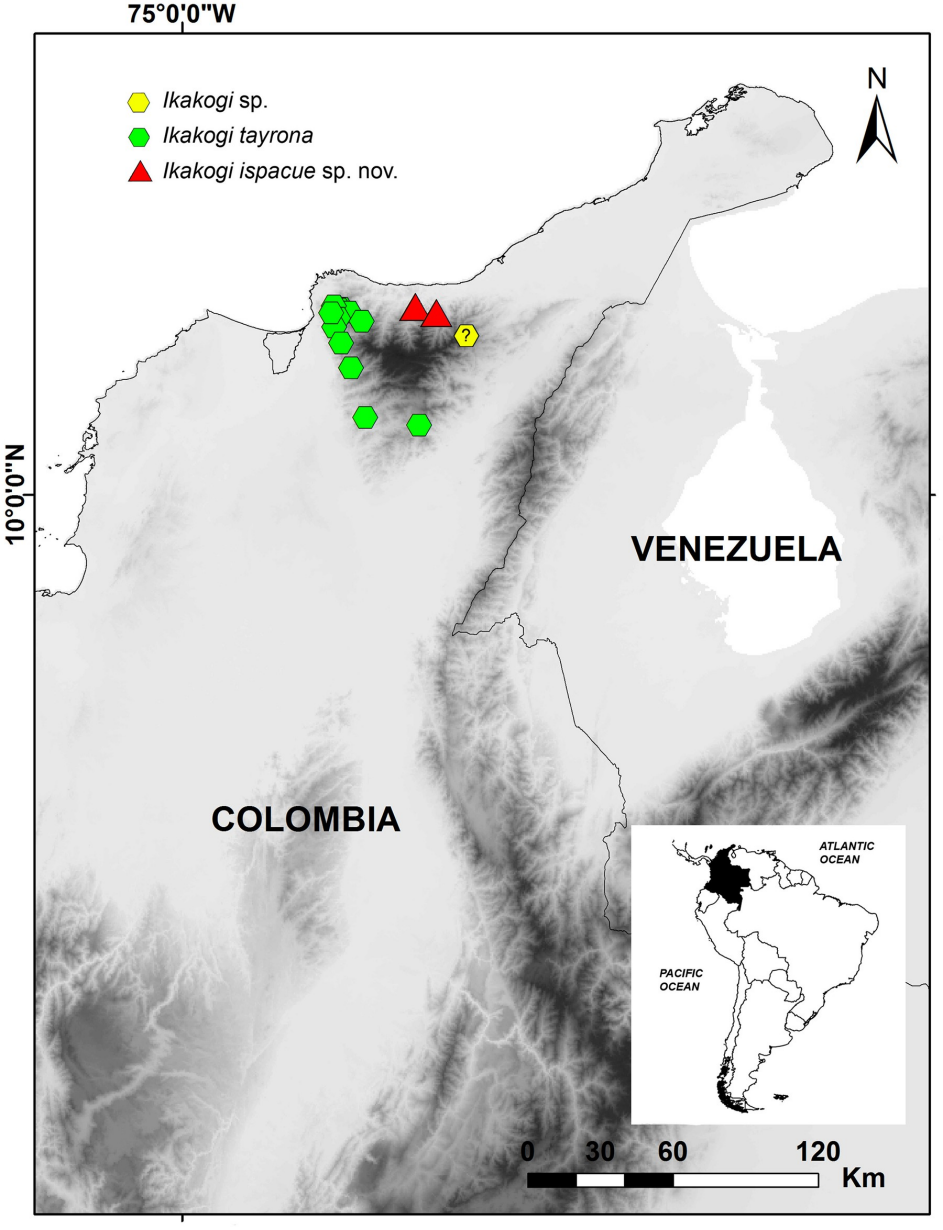
Map of northern Colombia showing locality records of *Ikakogi tayrona* and *I*. *ispacue* sp. nov. Detailed information about localities is provided in S2 Appendix.

**Fig 8:**
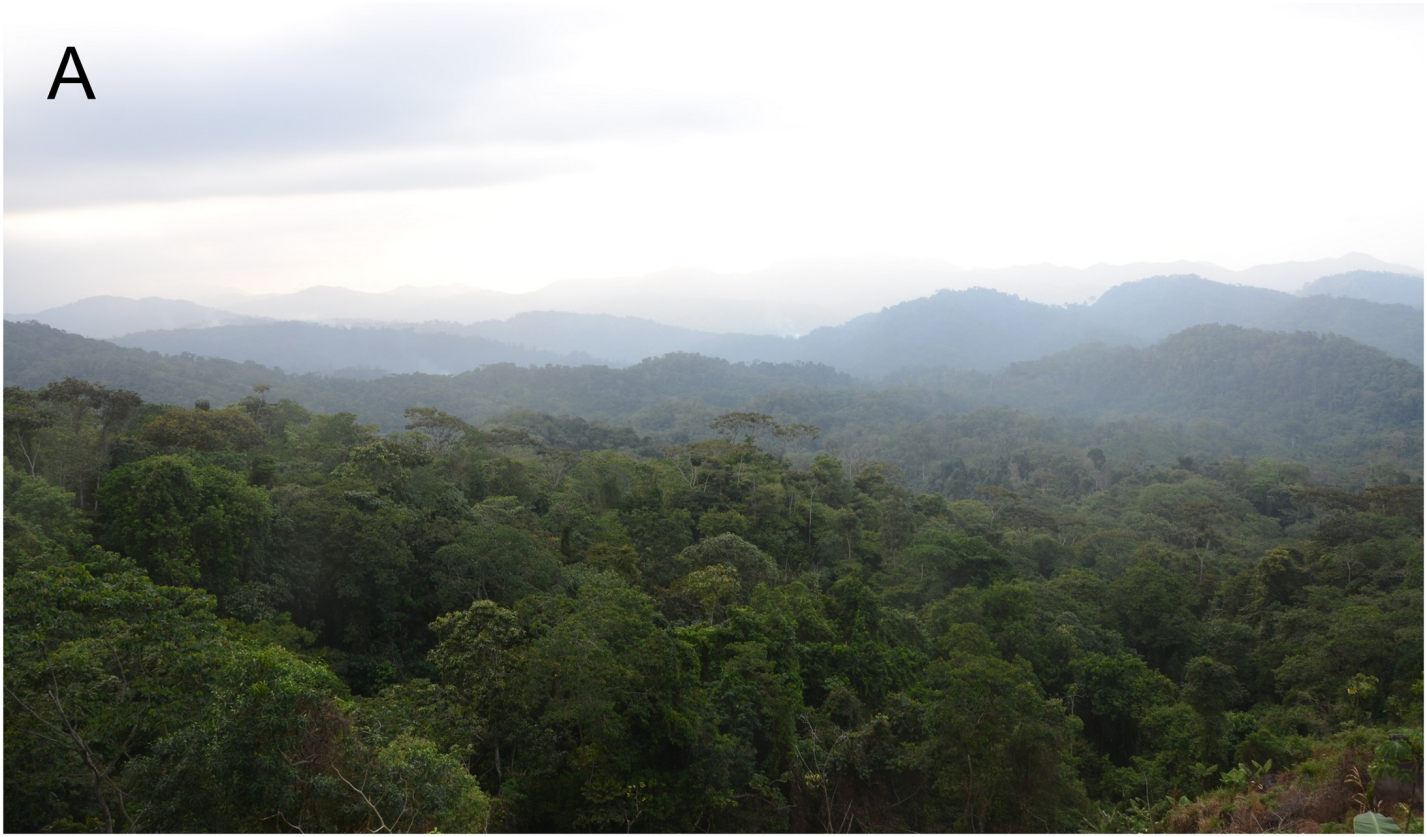
Type locality of *Ikakogi ispacue* sp. nov. Northern flank of Sierra Nevada de Santa Marta (April 1, 2015), general view from 950 m asl.

*Ikakogi ispacue* sp. nov. is currently known only from the type locality, a small stream tributary of the Río San Salvador on the northern flank of the Sierra Nevada de Santa Marta 11,12480556 / -73,55647222 at 950 m a.s.l and from Riohacha, Monte Cheturrycuak, sitio La Cueva, small stream on the headwater of Río Tapias, 11,058091/ -73.227770 at 850 m a.s.l (Figs 7 and 8). See S2 Appendix.

#### Natural history

Males and females were observed in subtropical forest perched on the vegetation at heights of 1–2 m. (Fig 4). Males were observed calling from the lower and upper surfaces of leaves along streams at height of 50–350 cm approx. (Fig 4A). Females deposited and cared for egg clutches (referred to the species by association with their mothers perched on clutches) on either the upper side or lower side of leaves overhanging streams (ca. 50–100 cm; Fig 4B). Clutches contained uniformly pale cream or pale green eggs (n = 4; 55 ± 6.21; 48–62 eggs; Fig 4B). The morphology of the egg mass observed near males of *I*. *ispacue* sp. nov. is a monolayer mass lacking eggs and jelly in the center of the clutch, which gives an appearance of a "ring" shape (n = 3; Fig 4C). Embryos exhibit cranial hypervascularization, which turned their color reddish or pink; the heart is translucent but colored reddish by blood. Tadpoles of *Ikakogi ispacue* sp. nov. were found buried in fallen leaves and sand in small pools (area = 1–2 m^2^; depth = 30–50 cm) located along the edge of streams.

#### Advertisement call

The call of the species consisted of a high-pitched note (“trill”) of 11–20 pulses with modulated amplitude (Fig 6) (n = 17; Table 3). Mean call duration was 0.122 ± 0.041 s (0.082–0.185 s) with a mean pulse duration of 0.007 ± 0.001 s (0.006–0.007 s); pulses were adjacent or were separated by silent intervals < 0.004 s in duration, although the terminal pulses in two calls were separated by 0.018 s (0.014–0.022 s) (Fig 6). Mean pulse rate was 137.25 ± 18.31 pulses/s (109.2–155.21 pulses/s). There was not a clear frequency modulation through the call; the dominant frequency of the whole call was 3129.37 ± 146.55 Hz (2928–3273 Hz), the lower frequency was 2646.73 ± 157.87 Hz (2440–2852 Hz), and the higher frequency was 3581.59 ± 85.23 Hz (3450–3685 Hz).

**Table 3:** Features of the advertisement call of five males of *Ikakogi ispacue* sp. nov. recorded at Río Ancho, Sierra Nevada de Santa Marta, Colombia.

Male	SVL (mm)	Temperature (°C)	Number of calls	Call duration (s)	Number of pulses	Pulse rate (pulses/s)	Mean Pulse duration (s)	Peak Frequency (Hz)	Low Frequency (Hz)	High frequency (Hz)
1	33.5	19.9	6	0.120	18	155.21	0.007	3072.07	2631.67	3568.33
2	32.7	20.6	5	0.088	11	129.16	0.007	3273.00	2852.00	3604.60
3	-	-	4	0.134	18	146.03	0.007	3273.00	2740.00	3685.00
4	-	-	1	0.185	20	109.20	0.006	2928.00	2440.00	3450.00
5	-	-	1	0.082	11	146.67	0.007	3100.80	2570.00	3600.00

### Larvae of Ikakogi ispacue sp. nov.

#### External morphology

**Fig 9:**
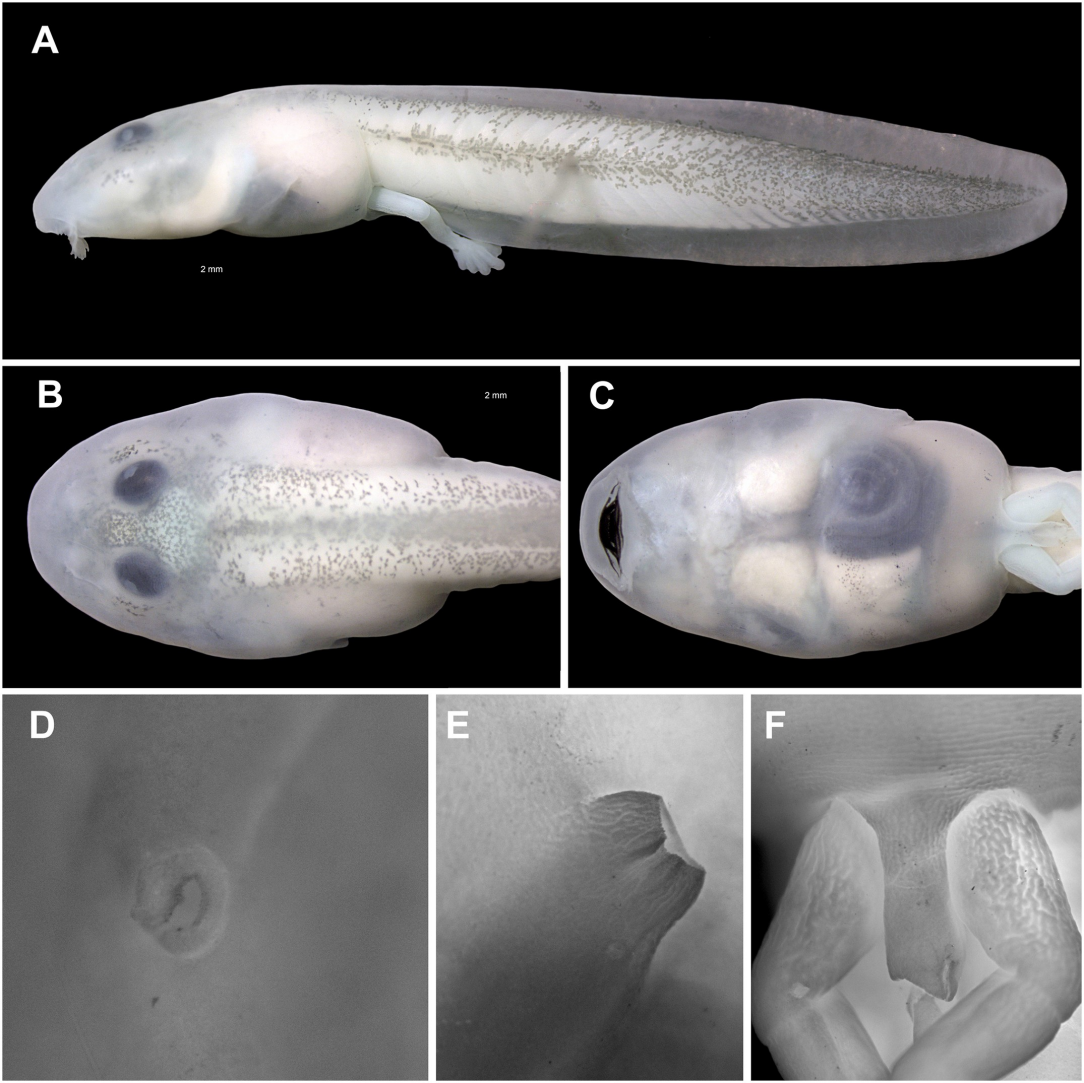
Tadpole of *Ikakogi ispacue* sp. nov. in lateral view. (A) Dorsal and ventral views (B–C). Note the short spiracle and the translucent venter (C), nostril, spiracle and vent tube respectively (D–F). CBUMAG:ANF 01015, stage 35. Scale bar equal to 2 mm (A–C).

**Fig 10:**
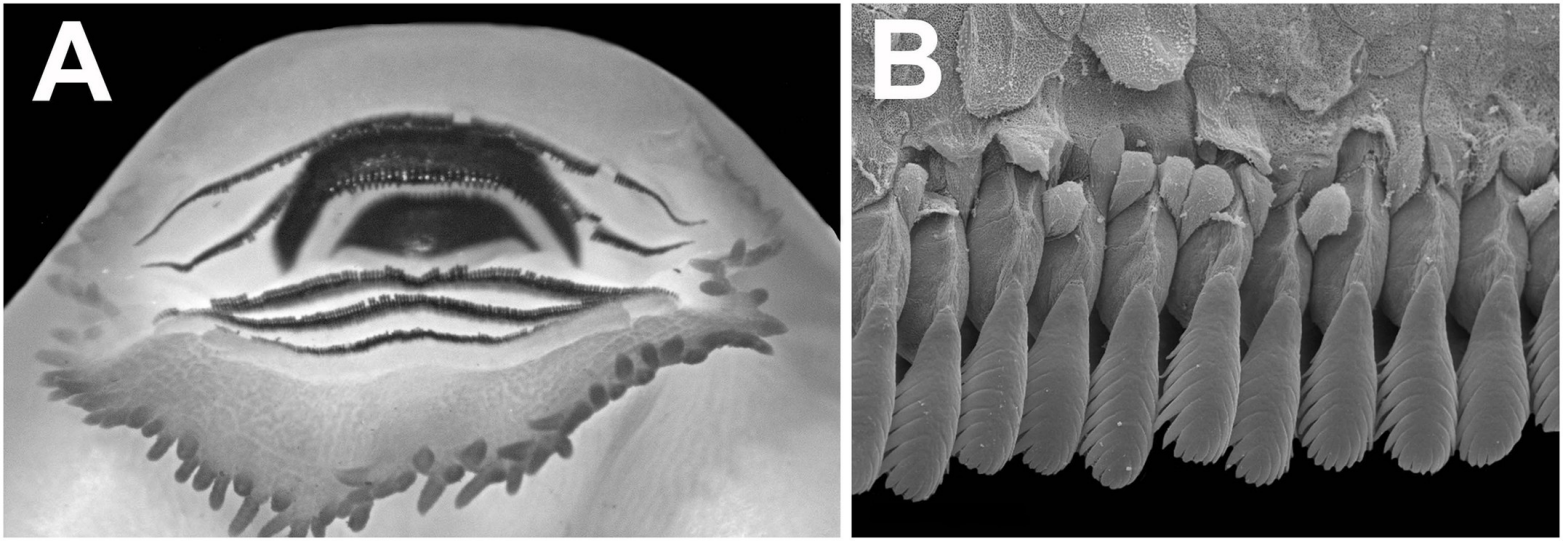
Oral disc of the tadpole of *Ikakogi ispacue* sp. nov. (A) and close-up of lateral teeth of upper jaw, stage 35; scale bar equal to 1.5 mm. (B). CBUMAG: ANF 01015, stage 35; scale bar equal to 20 μm.

All measurements are in mm (Figs 9 and 10; n = 1 at Gosner’s stage 25, CBUMAG: ANF 01015; Table 4). In dorsal view, body elongated, elliptical, wider medially, snout acuminated (Fig 9B). In lateral view, body elliptical, depressed (BH/BL = 41.1%), snout acuminated (Fig 9A). Eyes dorsal, reduced (ED/BW = 12.7%; ED/BL = 7.5%), directed anterolaterally. Nares reniform, located dorsally, directed anterolaterally; nares with marginal rim and small triangular fleshy projection on sagittal margin (Fig 9D). Interorbital distance 0.6 times eye diameter; IOD/IND = 63.6%.

**Table 4:** Mean and standard deviation of the morphological measurements (mm) of *Ikakogi ispacue* sp. nov. tadpoles.

Species	Stage	n	TL	BL	BW	BH	IND	IOD	SSD	ESD	MTH	TAL	TMH	ODW	VTL
*Ikakogi ispacue* sp. nov.	25	2	16,0 ± 0,6	4,6 ± 0,1	2,5 ± 0,1	1,5 ± 0,0	0,8 ± 0,1	0,9 ± 0,0	3,8 ± 0,1	1,4 ± 0,1	2,2 ± 0,1	11,4 ± 0,7	1,5 ± 0,1	1,2 ± 0,1	0,8 ± 0,0
			(15,6–16,5)	(4,5–4,7)	(2,4–2,5)	(1,5–1,5)	(0,7–0,9)	(0,8–0,8)	(3,7–3,9)	(1,3–1,5)	(2,2–2,3)	(10,9–11,9)	(1,5–16)	(1,2–1,2)	(0,8–0,8)
	26	7	7,0 ± 0,4	3,8 ± 0,1	2,3 ± 0,2	1,4 ± 0,1	1,1 ± 0,1	6,2 ± 0,3	2,5 ± 0,2	3,1 ± 0,3	17,1 ± 0,9	17,1 ± 0,9	2,1 ± 0,1	2,4 ± 0,2	0,8 ± 0,1
			(22,3–25,5)	(6,5–7,4)	(3,7–4,0)	(2,1–2,6)	(1,4–1,5)	(1,1–1,3)	(5,9–6,7)	(2,3–2,9)	(2,8–3,6)	(15,6–18,2)	(2,0–2,2)	(2,2–2,6)	(0,7–0,9)
	27	3	28,5 ± 0,8	8,4 ± 0,2	4,7 ± 0,2	2,7 ± 0,4	1,6 ± 0,2	1,2 ± 0,0	7,0 ± 0,3	2,9 ± 0,1	3,9 ± 0,3	20,1 ± 0,6	2,6 ± 0,11	2,8 ± 0,0	1,1 ± 0,0
			(27,6–29,0)	(8,2–8,6)	(4,5–4,9)	(2,3–3,2)	(1,4–1,9)	(1,9–1,2)	(6,7–7,3)	(2,8–3,0)	(3,7–4,1)	(19,5–20,5)	(2,5–2,7)	(2,8–2,9)	(1,0–1,1)
	28	2	30,4 ± 1,2	8,8 ± 0,4	5,3 ± 0,1	3,3 ± 0,8	2,0 ± 0,0	1,3 ± 0,0	7,6 ± 0,2	3,1 ± 0,3	4,2 ± 0,0	21,6 ± 0,8	2,6 ± 0,2	2,3 ± 0,1	1,1 ±0,0
			(29,6–31,2)	(8,5–9,1)	(5,2–5,4)	(3,8–2,8)	(2,0–2,0)	(1,3–1,4)	(7,4–7,8)	(2,9–3,3)	(4,2–4,2)	(21,0–22,2)	(2,5–2,7)	(2,71–2,8)	(1,2–1,1)
	35	1	36,4	10,7	6,3	4,4	2,2	1,4	9,0	3,3	5,0	25,7	3,4	3,0	1,2

Mean ± standard deviation; range into parenthesis. Meristic measurements were: (TL) total length; (BL) body length; (BW) body width; (BH) body height; (IND) internarial distance; (IOD) interorbital distance; (SSD) spiracle-snout distance; (ESD) eye-snout distance; (TAL) tail length; (ESD) eye-snout distance; (MTH) maximum tail height; (TMH) tail muscle height; (ODW) oral disc width; (VTL) vent tube length.

Mouth anteroventral (Fig 9C), not emarginated laterally, bordered by single row of 64 conical, alternated, marginal papillae; upper lip with large diastema; marginal papillae longest on the medial lower lip; submarginal papillae absent (Fig 10A); oral disc 47.6% of body width. Labial tooth row formula 2(2)/3; A-1 = A-2, P-1 = P-2>P-3; A2 gap large, extending through the descending border of the upper jaw. Jaw sheaths present, serrate, keratinized; upper jaw sheath arch-shaped; lower jaw sheath U-shaped; upper jaw sheath wider than lower jaw sheath (Fig 10A). Labia tooth wider distally, body narrow; head convex, cuspidate; tooth body-head with medial constriction; 18–20 cusps on each tooth (Fig 10B).

Spiracle sinistral, tubular, short, posterolaterally located, at 84.1% of body length (BL), directed posterolaterally in dorsal view, dorsad at angle of 30–45° in lateral view; inner wall present, distally free from the body, longer than external wall; opening elliptical (Fig 9E). Vent tube medial, tubular, positioned at the level of ventral fin, fused to ventral fin medially (Fig 9C and 9F). Tail long (TAL/TL = 70,6%), low (BH/MTH = 88%); caudal muscles not reaching rounded tip; dorsal fin arched, originating on body/tail junction, ventral fin arched; dorsal fin slightly higher than ventral fin. Myotomes V-shaped, arranged in serial blocks; maximum tail height 19.3% of total length. Lateral line stitches conspicuous, including X, Y and Z lines.

#### Measurements

TL = 36.4; BL = 10.7; BW = 6.3; BH = 4.4; DFH = 0.9; VFH = 0.9; ED = 0.8; IND = 2.2; IOD = 1.4; SSD = 9.0; ESD = 3.3; MTH = 5.0; TMH = 3.0; TMW = 2.6; TAL = 25.7; ODW = 3.0; VTL = 1.2.

#### Color in life

High-vascularized skin, which gives the larvae a reddish or pinkish general appearance. Dorsum slightly pigmented but with some minute dark gray scattered punctuations especially concentrated between the eyes, in the posterior section of body and along the tail. Ventral skin of body translucent, intestine translucent, liver dark red, heart and other parts of the circulatory system are bright red. Tail musculature reddish with conspicuous brownish longitudinal stripe in the midline of myotomes, extending from the tail-body junction until almost 1/ 3 of the tail. Tail fins are transparent with minute melanophores mainly in the distal portion.

#### Color in preservative

The color pattern is similar to that of living tadpoles but loses its red or pink coloration. The dorsum, tail musculature, and venter are light cream. Melanophores on dorsum, tail fins and tail musculature turn pale gray (Fig 9A–9F).

#### Variation and ontogenetic development

Variation of 13 meristic characters of tadpoles in stages 25–35 are given in Table 4. Labial tooth row formula, 2(2)/3, invariable at different stages (Table 5); nevertheless, at stage 25 the upper and lower tooth rows are less developed, i.e., teeth are weakly keratinized and interrupted in some areas. Extension of middle dark gray stripe of the tail musculature slightly varies between the half and posterior third. In some specimens the tail tip is bluntly pointed or rounded (Fig 9A). In stages 25 to 28, the eyes of *Ikakogi ispacue* sp. nov. are small, scarcely pigmented and C-shaped in dorsal view, whereas they are larger and more pigmented and round by stage 35.

**Table 5:** Summary of external morphological characters and oral apparatus found in tadpoles of different species of Centrolenidae.

Species	Stage	LRTF	Upper jaw sheath shape	Snout–Spiracle/body distance	Snout shape (lateral view)	Dorsal coloration in preservative	Reference
*C*. *altitudinale*	25	1/2–3	Inverted U–shaped*	Posterior (74–81%)	Rounded*	Slightly pigmented	[48]
*C*. *daidaleum*	24	0/0	Inverted U-shaped**	Posterior (72%)*	Acuminated	Slightly pigmented*	[49]
	25	1/2; 2/2		Posterior (88%)**			
	26–27	2/2		Posterior (82–84%)**			
	28	2/2; 2/1		Posterior (71–79%)**			
	36	2(2)/2*		Posterior (78%)			
	41	0/0		Posterior (85%)**	Truncated		
*C*. *hesperium*	25	2(2)/3	–	Posterior	Rounded	–	[44]
*C*. *savagei*	25	–	–	Posterior (85%)*	–		[59]
	26	2(1)/3	Inverted U-shaped**	Posterior (87%)*	Rounded	Slightly pigmented	
	28–30	–	–	Posterior (80%)*	–		
	39	1(1)/2(2)	Inverted U-shaped**	Posterior (81%)	Rounded	Slightly pigmented	
*C*. *revocata*	25	1/3	Inverted U-shaped*	Posterior (84%)*	Rounded*	Slightly pigmented*	[48]
*C*. *vozmedianoi*	25	1/3	Inverted U-shaped*	Posterior (71–93%)*	Rounded*	Slightly pigmented*	[48]
*C*. *euknemos*	25	2(2)/3*	M-shaped**	Posterior (64%)*	Rounded	Slightly pigmented	[51]
	34 and 39	–	–	Posterior (65%)*	Rounded and Acuminated**	Slightly pigmented	
*C*. *granulosa*	25	2(2)/3*	M-shaped*	Posterior (66%)*		Strongly pigmented*	[40]
	27–28				Rounded	Slightly pigmented*	[51]
*C*. *guayasamini*	26	1/3					[11]
	35		–	–	–	Slightly pigmented*	
	39						
*C*. *resplendens*	24	0/0				Slightly pigmented	[54]
	25–38	2(2)/3	Inverted U-shaped*	Posterior (69–75%)*	Rounded		
	42	1/0					
*E*. *andina*	25	1/3	M-shaped	Posterior*	Rounded	Strongly pigmented/Slightly pigmented	[38]
	36	2(2)/2(1)		Posterior (75–83%)			[48]
*E*. *prosoblepon*	–	2(2)/3*	Inverted U-shaped*	Posterior	Rounded	Slightly pigmented*	[40]
	27		–	Posterior (79%)*	Acuminated*	Strongly pigmented/Slightly pigmented*	[51]
*H*. *aureoguttatum*	23	1/1	–	–	Rounded	Slightly pigmented	[54]
	24–35	2(2)/3	M-shaped	Posterior (67–79%)*			
*H*. *cappellei*	25	0/2	–	–	–	–	[45]
	25	–	Inverted U-shaped*	Posterior (69–71%)	Rounded*	Slightly pigmented*	[48]
	25	0/3	Inverted U-shaped**	Posterior	Acuminated**	Slightly pigmented*	[47]
*H*. *chirripoi*	25–41	2(2)/3*	Inverted U-shaped*	Posterior*	Rounded	Slightly pigmented*	[51]
*H*. *colymbiphyllum*	25	2(2)/2–3	Inverted U-shaped**	Posterior (70%)*	Rounded*	Strongly pigmented*	[46]
	27	2(2)/3(1) *	Inverted U-shaped*	Middle (53%)	Rounded	Slightly pigmented*	[51]
*H*. *duranti*	–	0/3**	Inverted U-shaped*	Middle (50%)/ Posterior (68%)*	Rounded*	Slightly pigmented*	[48]
*H*. *fleischmanni*	–	2(2)/3**	Inverted U-shaped*	Posterior*	Rounded**	Slightly pigmented*	[40]
	25–39	2(2)/3*	Inverted U-shaped**	Posterior (72%)*	Rounded**	Slightly pigmented*	[51]
*H*. *ibama*	24	0/0	–	Middle (58%)**	Rounded*	Slightly pigmented*	[49]
	25	1/1; 2/0; 2/2 and 2/1–2/3	–	Posterior (60%)**			
	26	2/2–2/3	–	Middle (58–59%)**			
	27–29	2(2)/3*	–	Middle (55%)/ Posterior (71%)**			
	31	2/2	–	Middle (59%)**			
	36	2/2	Inverted U-shaped*	Middle (57%)			
	41	2/1	–	Posterior (66%)**	Truncated		
	42	0/0	–	Middle (59%)**	–	Greenish**	
*H*. *orientale*	25	1/3	M-shaped	Middle (46–55%)	Rounded*	Slightly pigmented*	[48]
*H*. *orientale tobagoense*	25	2(2)/3(1)	–	Middle (58%)	Rounded	Slightly pigmented*	[55]
*H*. *talamancae*	25–29	2/3	Inverted U-shaped*	Middle (53%)	Rounded	Slightly pigmented*	[51]
*H*. *taylori*	25	1/3	M-shaped	Posterior (75–84%)	Rounded*	Slightly pigmented*	[48]
*H*. *valeroi*	–	2(2)/3*	M-shaped**	–	–	Slightly pigmented*	[40]
	25–41		M-shaped	Posterior (65–71%)*	Rounded		[51]
*H*. *vireovittatum*	25	2/3	Inverted U-shaped*	Middle (57%)	Rounded	Slightly pigmented*	[51]
*I*. *ispacue* **sp. nov**.	25–35	2(2)/3	Inverted U-shaped	Posterior (63–81%)	Acuminated	Slightly pigmented	This study
*I*. *tayrona*	–	2(2)/3**	Inverted U-shaped**	Posterior*	Acuminated**	Slightly pigmented**	[41]
	24–34			Posterior (60–83%)**			This study
	35–39	2(2)/2**		Posterior (68–83)**			
	40	2(2)/2; 1(1)/2		Posterior (82%)**			
	41	2(2)/1; 1(1)/2 and 1/1		Posterior (71–81%)**	Rounded		
	42	0/2		Posterior (78)**	Truncated	Greenish**	
	43	0/0		Posterior (68–70)**	Truncated	Greenish**	
*N*. *grandisonae*	24	0/0–2/2–2/3		Posterior (68–70%)*		Slightly pigmented*	[52]
	25–27	2/2		Posterior (60%)*			
	28–29	2/2–2/3		Middle (55%)/ Posterior (61%)*	Rounded**		
	32	2/3	M-shaped	Posterior (64%)*			
	36	2(2)/3*		Posterior (60%)*	Rounded		
	38	2/2–1/2		Posterior (63%)*			
	39	1/2		Posterior (60%)*			
*S*. *albomaculata*	25–37	–	Inverted U-shaped*	Posterior (67–81%)*	Rounded*	Strongly pimented	[51]
*S*. *ilex*	25–36	–	Inverted U-shaped*	Posterior (75–81%)*	Acuminated*	Strongly pimented*	[51]
*T*. *pulverata*	24–41	2/3	Inverted U-shaped*	Posterior (65–78%)*	Acuminated**	Strongly pimented*	[81]
	25–39	–	Inverted U-shaped*	Posterior (70%)	Rounded**		[51]
*T*. *spinosa*	–	0/3**	Inverted U-shaped*	Posterior*	Rounded**	Strongly pimented*	[40]
	25–36	–		Posterior (77–78%)*	Acuminated*		[51]
*V*. *castroviejoi*	25	1/3	Inverted U-shaped*	Middle (53–57%)*	Rounded*	Slightly pigmented	[48]
*V*. *eurygnatha*	25–42	–	Inverted U-shaped**	Posterior	Acuminated**	Slightly pigmented*	[42]
	28 and 35	0/0					This study
*V*. *helenae*	25	1/3	Inverted U-shaped*	Posterior (75–80%)*	Rounded	Strongly pigmented	[48]
*V*. *ritae*	25	0/1–2	–	–	–	Slightly pigmented*	[43]; [48]
	25		Inverted U-shaped**	Posterior	Acuminated**		[50]
*V*. *uranoscopa*	25–41	1/2 1/1 1/3	Inverted U-shaped**	Posterior	Acuminated**	Slightly pigmented*	[42]
	31	2/2					This study

A single asterisk (*) denotes additional information or some modification of the character/state from the original source.

Double asterisks (**) denote new information.

#### Internal morphology

**Fig 11:**
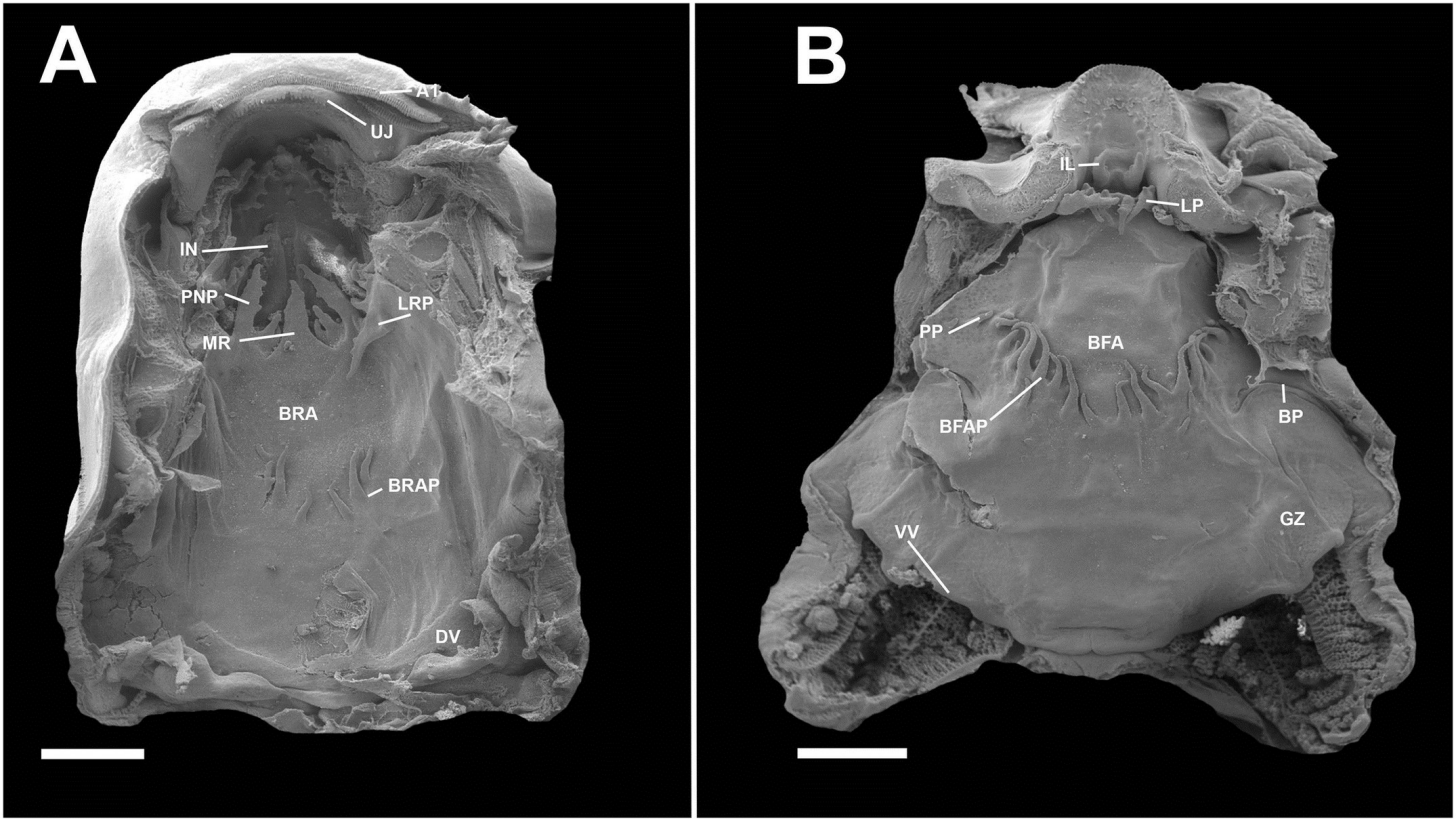
Buccopharyngeal morphology of the tadpole of *Ikakogi ispacue* sp. nov. Buccal roof (A), and buccal floor (B). Abbreviations: A1, first anterior tooth row; BFA, buccal floor arena; BFAP, buccal floor arena papillae; BP, buccal pocket; BRA, buccal roof arena; BRAP, buccal roof arena papillae; DV, dorsal velum; GZ, glandular zone; IL, infralabial papillae; IN, internal nares; LP, lingual papillae; LRP, lateral ridge papillae; PNP, postnarial papillae; PP, prepocket papillae; UJ, upper jaw sheath; VV, ventral velum. CBUMAG: ANF 01016, stage 28. Scale bars equal to 500 μm.

**Fig 12:**
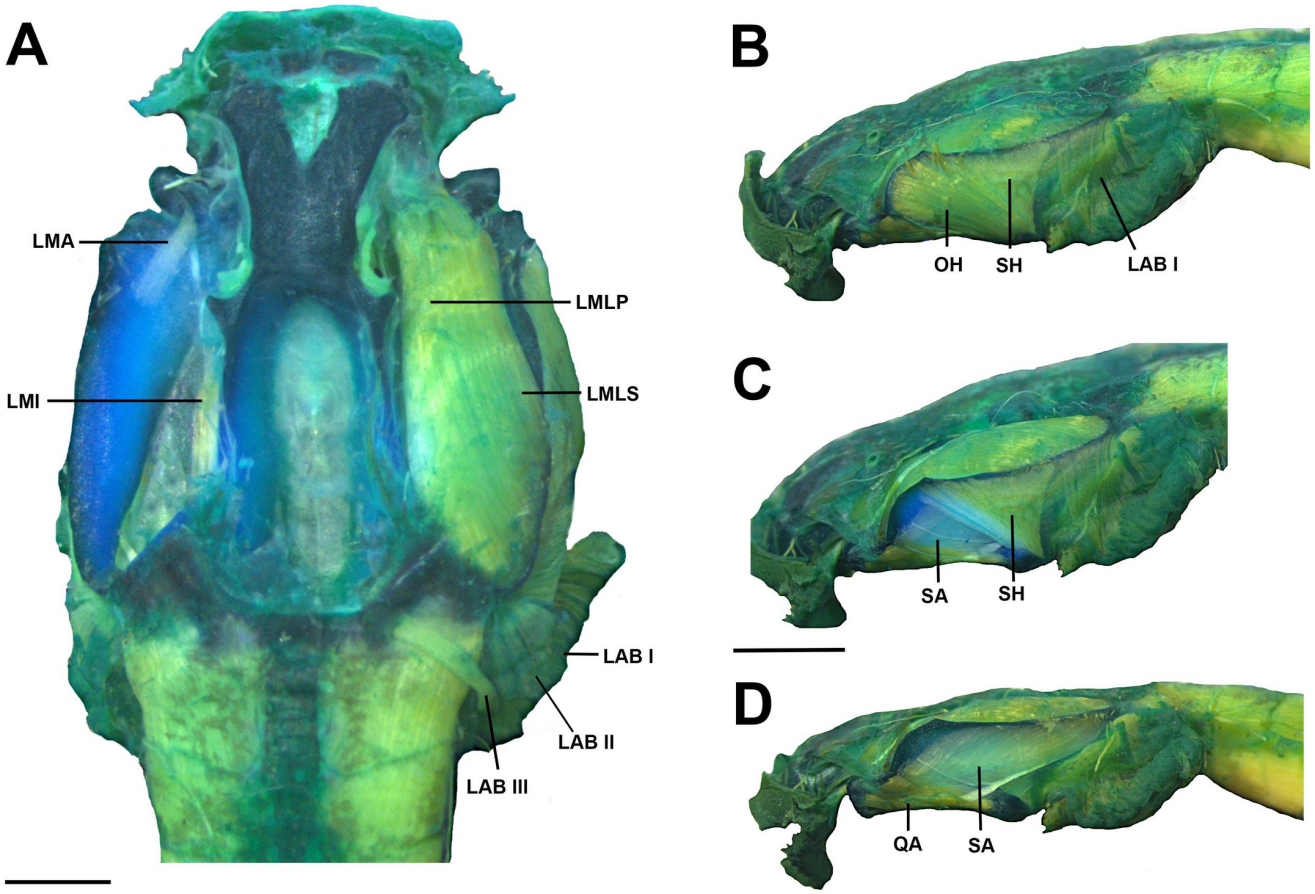
Cranial muscles of the tadpole of *Ikakogi ispacue* sp. nov. Dosrsal (A) and lateral views (B–D). Abbreviations: LAB (I to III), *musculus levator archus branchialum* I to III; LMA, *m*. *levator mandibulae articularis*; LMI, *m*. *levator mandibulae internus*; LMLP, *m*. *levator mandibulae longus profundus*; LMLS, *m*. *levator mandibulae longus superficialis*; OH, *m*. *orbitohyoideus*; QA, *m*. *quadratoangularis*; SA, *m*. *suspensorioangularis*; SH, *m*. *suspensoriohyoideus*. CBUMAG: ANF 01016, stage 28. Scale bar equal to 1.0 mm.

**Fig 13:**
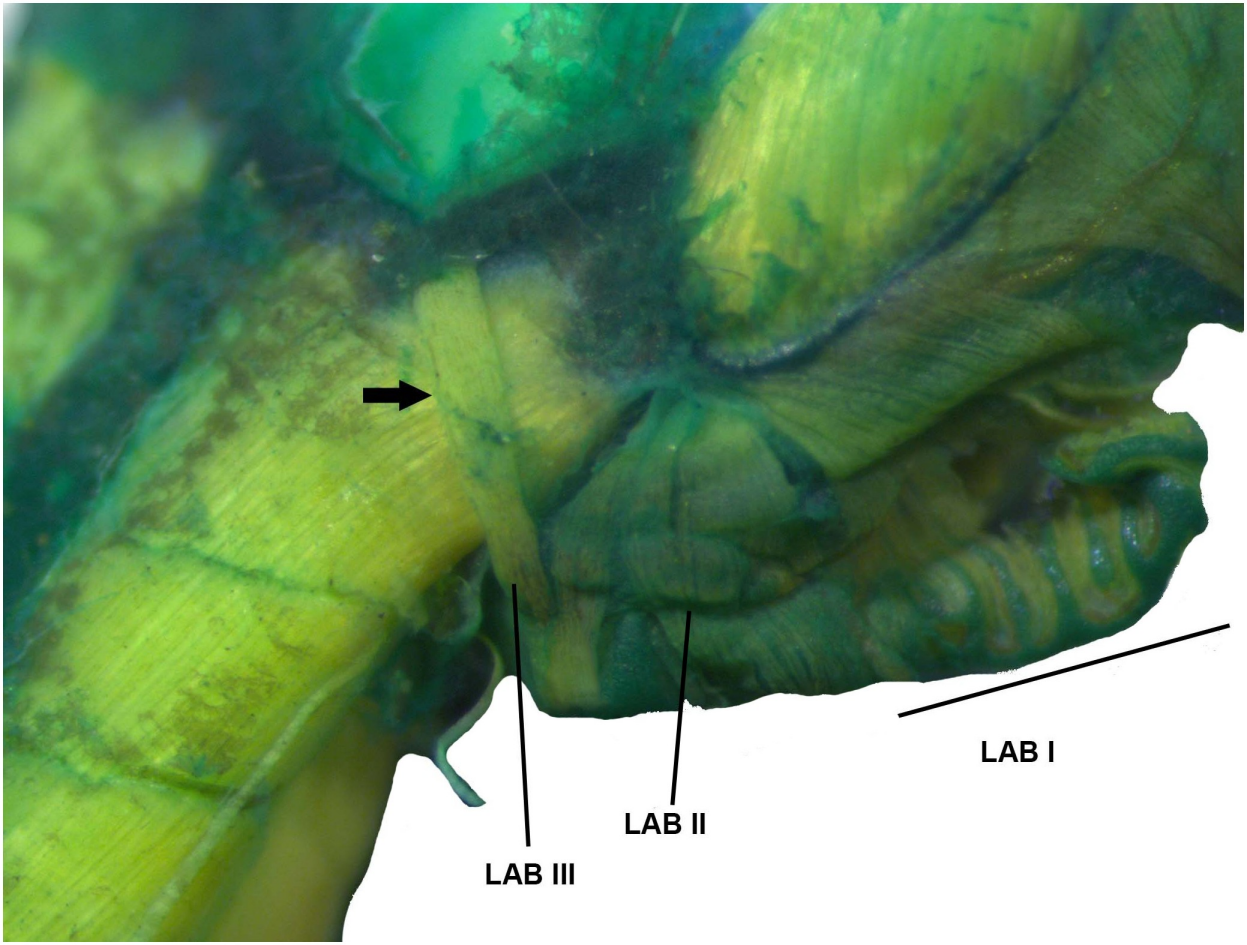
Detail of the musculus levator archus branchialium (LAB) in tadpoles of *Ikakogi ispacue* sp. nov. Arrow indicates origin in dorsal otic capsule. CBUMAG: ANF 01016, stage 28. Scale bar equal to 1.0 mm.

**Fig 14:**
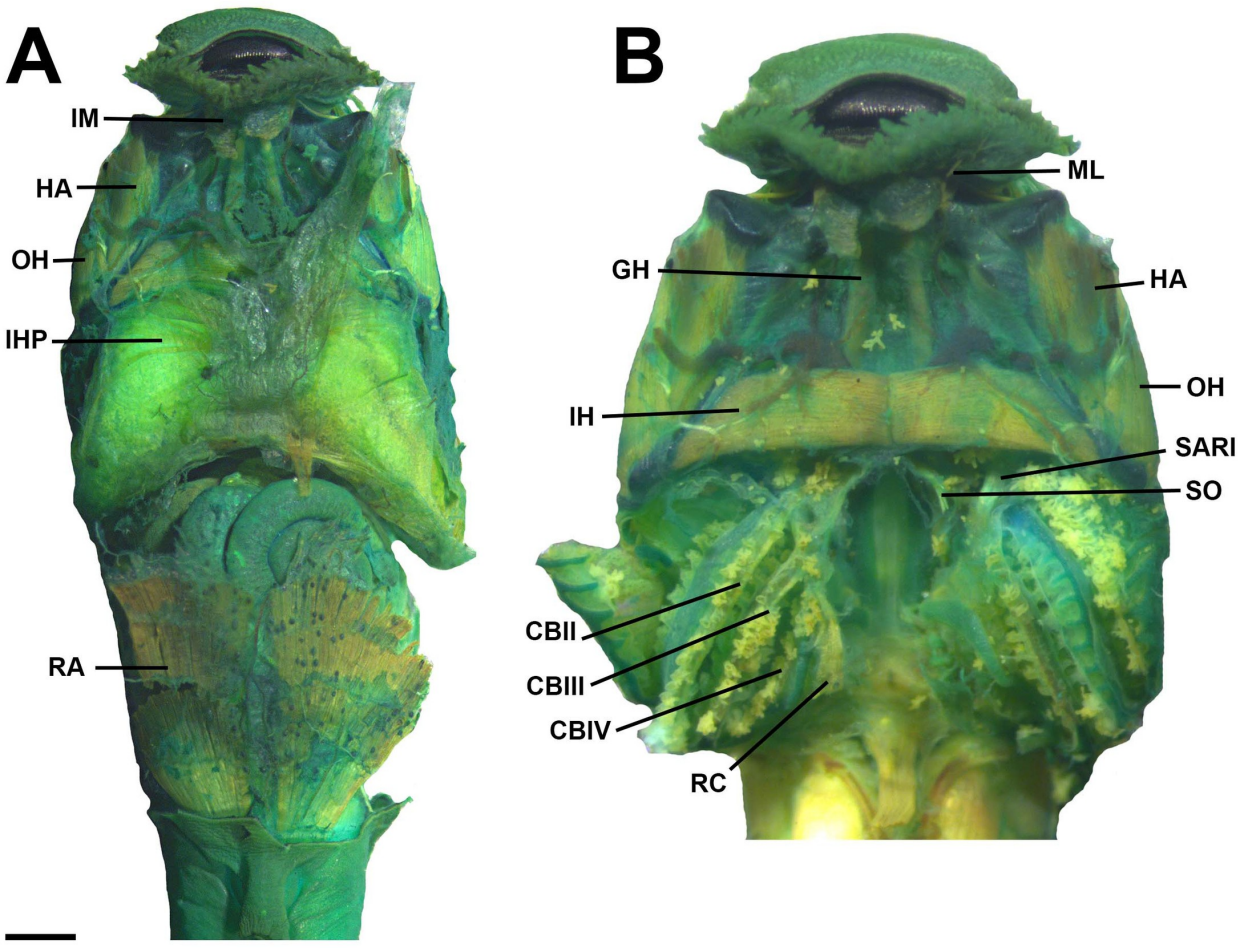
Cranial muscles of the tadpole of *Ikakogi ispacue* sp. nov. in ventral view (A–B). Abbreviations: CB (II-IV), *musculus constrictor branchialis* II to IV; GH, *m*. *geniohyoideus*; HA, *m*. *hyoangularis*; IH, *m*. *interhyoideus*; IHP, *m*. *interhyoideus posterior*; IM, *m*. *intermandibularis*; OH, *m*. *orbitohyoideus*; RA, *m*. *rectus abdominis*; RC, *m*. *rectus cervicis*; SAR I, *m*. *subarcualis rectus* I; SO, *m*. *subarcualis obliquus*. CBUMAG: ANF 01016, stage 28. Scale bar equal to 1.0 mm.

**Fig 15:**
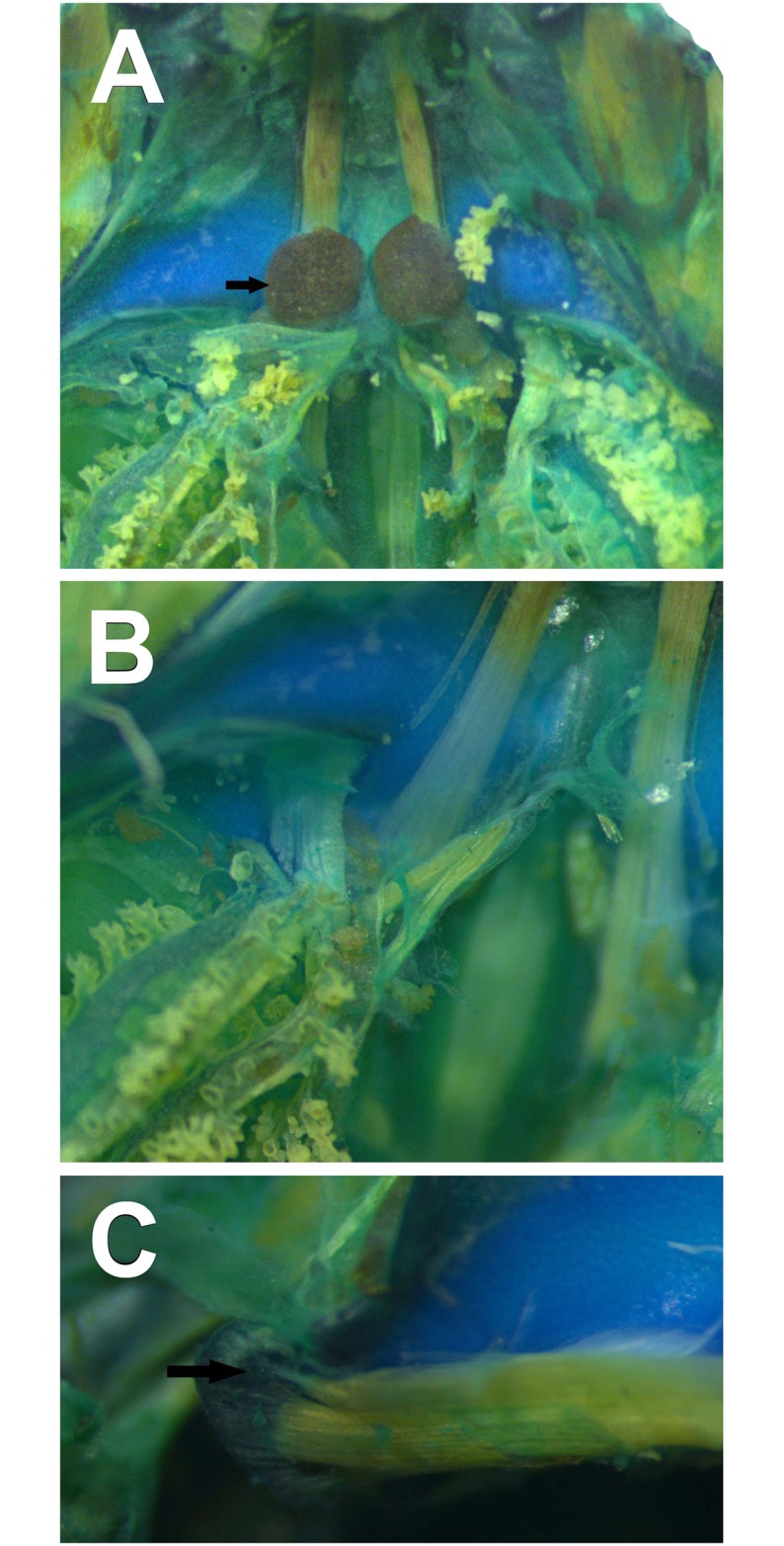
Detail of the sinus hyobranchialis of the tadpole of *Ikakogi ispacue* sp. nov (arrow). (A). Detail of the musculus subarcualis obliquus with two slips (B), and m. hyoangularis with two slips (C); arrow indicates the small tendon. CBUMAG: ANF 01016, stage 28. Scale bar equal to 1.0 mm.

**Fig 16:**
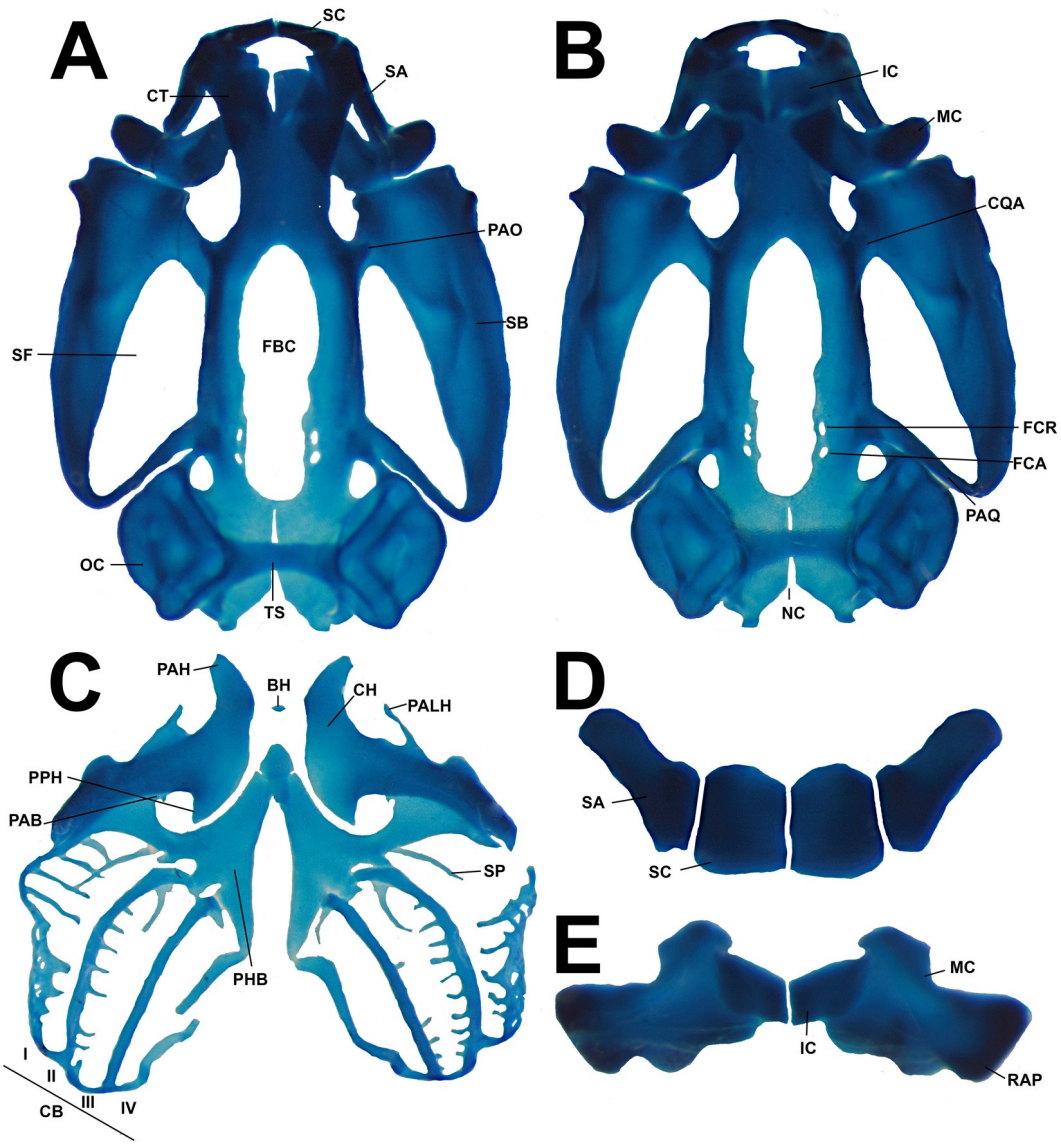
Larval chondrocranium morphology of the tadpole of *Ikakogi ispacue* sp. nov. Dorsal (A), and ventral views (B), apparatus hyobranchialys (C), suprarostral (D) and infrarostral cartilage and Meckel`s cartilage (E). CBUMAG: ANF 01016, stage 28 Scale bar equal to 1 mm. Abbreviations: BH, basihyal; CB (I-IV), ceratobranchials I to IV; CH, ceratohyal; CQA, commissura quadratocranialis; CT, cornua trabeculae; FBC, fenestrae basicranialis; FCA, foramen caroticum primarum; FCR, foramen craniopalatinum; IC, infrarostral cartilage; Meckel`s cartilage; NC, notochord canal; PAB, processus anterior branchialis; PAH, processus anterior hyalis; PALH, processus anterolateralis hyalis; PAO, processus antorbitalis; PHB, planum hypobranchialis; PPH, processus posterior hyalis; RAP, retroarticular process of Meckel`s cartilage; SA, suprarostral ala; SB, subocular bar; SC, suprarostral corpora; SF, subocular fenestrae; SP, spicules. Scale bar equal to 1.0 mm.

Figs 11–16, n = 2 at Gosner’s stage 28 and 35, CBUMAG: ANF 01015.

#### Buccopharyngeal cavity—(Fig 11A and 11B)

Buccal roof elongated, elliptical, longer than wide, with most features concentrated in the anterior portion. Prenarial arena semi-circular, with inverted V-shaped dermal crest covered with pustulations. Internal nares elliptical, oriented vertically, longitudinally to body`s main axis; anterior and posterior margins with large conical projections; valve projection present, triangular, well developed, positioned laterally. Vacuities present, circumscribed by the inner nares margins. Postnarial papillae present, conical, arranged in series of three; third postnarial papillae is the tallest, bearing pustulations, located laterally to median ridge. Median ridge conical, tall. Lateral ridge papilla present, simple, long, triangular, with small pustulations; three triangular papillae present near the lateral ridge papillae. Buccal roof arena poorly delimited, U-shaped, lacking pustulations; five tall, conical papillae present in the buccal roof arena, the anterior ones laterally located, the posterior two, medially. Dorsal velum arched, devoid of papillae, medially continuous (Fig 11A). Buccal floor wider posteriorly than anteriorly, with most features concentrated in the anterior portion. Infralabial papillae present in two pairs; central pair shorter, conical, bearing postulation; lateral pair large tall, located on Meckel`s cartilage, wider on the base, bearing pustulations. Few pustulations on mouth`s opening. Lingual bud poorly discernible, bearing four lingual, conical, tall, papillae. Prepocket papillae present. Buccal pocket poorly marked superficially;circularly perforated. Buccal floor arena U-shaped, devoid of pustulations; ten lateral buccal floor arena papillae conical, tall, investing the central arena (one of those papillae is a four branched, large papilla). Glandular zone well marked; spicular support evident. Ventral velum well marked, medially continuous, with marginal projection, and discrete medial notch; ventral surface with glandular porous (Fig 11B).

#### Larval muscles

The *musculus interhyodeus posterior* and the *m*. *diaphragmatoparaechordalis* are present Figs 12–15; S5 Appendix; the former is well developed and continuous medially (Fig 14A). Jaw muscles well developed; the *m*. *levator mandibulae longus profundus et superficialis* are massive, occupying the entire area of the fenestra sub-ocular (Fig 11A). Laterally, the origin of the *m*. *suspensorioangularis* occupies the entire lateroventral surface of the palatoquadrate (Fig 11D). Large axial muscles, inserting on the anterior portion of the dorsal otic capsule. The most striking muscle is the well-developed *levator arcus branchialium III* with two slips: one originating in the dorsal otic capsule and crossing the axial muscles to insert in the ceratobranchial III (Fig 13), and the other on originating on posterior palatoquadrate and ventrolateral otic capsule.

#### Sinus hyobranchialis

The *sinus hyobranchialis* lies ventrally to the hyobranchial apparatus [81, 51]. In *Ikakogi* larvae the *sinus* is reduced and restricted to the basic *receptaculum disci oralis* (RDO), lacking the *receptaculum lateralis et transversalis*. The RDO is rounded and small, located at the level of the *processus urobranchialis* of the ceratohyal; in preserved specimens a mass of blood tissues can still be observed. The *vena lateralis hyobranchialis* is well developed and large in diameter. Both individuals possess slender guts and lack lungs (Figs 14 and 15).

#### Skeleton

*Chondrocranium* completely cartilaginous, almost ovoid, and broad posteriorly (at the level of the posterior palatoquadrate) (Fig 16A and 16B). Rostral region slightly depressed. In lateral view, the highest part of the *chondrocranium* at the ceratohyal plane and muscular process (Fig 16A). Suprarostral cartilage quadripartite, consisting of a central *corpus*, laterally in contact to the lateral alae (Fig 16A). Central *corpus* formed by two robust rectangular plates, articulated medially along most of their extension. Triangular-shaped alae, robust, posterodorsal processes with rounded tip. Suprarostral articulates with the trabecular horns through the *corpus* and alae regions. Trabecular horns large (45–48% of the *chondrocranium* length), slightly curved ventrally; anterior margin rounded with a medial projection, inner and outer margins smooth. Lateral trabecular process indistinct. Nasal structures not visible.

In the cranial floor, basicranial fenestra slightly occluded by a thin translucent cartilage, the *intertrabeculare planum*, pierced by two rounded pairs of foramina. The posterior, the primary carotid and the anterior and smaller, the craniopalatine foramen (Fig 16A and 16B). Lateral walls of the braincase formed by translucent orbital cartilages, extended towards the otic capsule, joined by a thin bar of cartilage arising from base of the pila antotica. Optic and oculomotor foramina visible on the posteroventral region of the orbital cartilage; oculomotor foramen open dorsally. Large frontoparietal fontanelle, approximately 70–75% of the *chondrocranium* length, almost rectangular (less width anterior than posteriorly), laterally bordered by the translucent orbital cartilage (there is no *taeniae tecti marginales*), anteriorly by the posterior trabecular horns and posteriorly by a thin *tectum synoticum*. *Taeniae tecti medialis* and *transversalis* absent. Otic capsules square-shaped, well developed and robust, 22–25% of the *chondrocranium* length. Anterolateral process of the crista parotica indistinct; larval otic process absent. Large oval fenestra. Jugulare and inferior perilymphatic foramina present, both visible from ventral view of the otic capsules.

Palatoquadrate wider anteriorly than posteriorly, their posterior regions curved to the *chondrocranium* (Fig 16B). Short articular process, projected anteriorly to articulate with the Meckel’s cartilage. Thin quadratocranial commissure, joining the palatoquadrate with the neurocranium. Quadratoethmoid and pseudopterygoid processes of the quadratocranial commissure absent. Muscular process of the quadratocranial commissure very low, its rounded tip oriented vertically, not surpassing the antoorbital process. Hyoquadrate process notable. Outer margin of the palatoquadrate slightly raised, directed laterally in lateral view. Palatoquadrate and *neurocranium* connected posteriorly through the ascending process, a rod-like cartilage, directed anteromedially and attached posteroventrally to the oculomotor foramen [82, 63]. The posterior curvature of the palatoquadrate extends to the anterior level of the capsula auditiva.

In the lower jaw (Fig 14B and 14E), sigmoid Meckel’s cartilages oriented almost perpendicular to the main axis of the *chondrocranium*, ventral to the trabecular horns. Meckel`s cartilage articulates to the articular process of the palatoquadrate by the posterior surface of the very short retroarticular process and opposite internal margin of Meckel’s cartilage (Fig 16E); dorsomedial and ventromedial processes of the cartilague of Meckel’s very low. Infrarostral cartilages rectangular, lateral ends thinner than medial region, joined medially by connective tissue, describing a weak wide V-shape. Small ovoid *corpus* cartilages between the ceratobranquial and palatoquadrate absent.

In the hyobranchial apparatus (Fig 16C), ceratohyals flat, well extended laterally and projected anteriorly by mean of the sub-acuminate anterior process, and posteriorly by a notable and almost triangular posterior process (see Fig 16C). Anterolateral process notable, tip subacuminated. Ceratohyals joined by the partially chondrified pars reuniens. Basihyal (copula I) evident. Basibranchial diamond-shaped, bearing a low and truncate urobranchial process, fused to the hypobranchial plates (two very long thin sheets not articulated medially). Outer margin of hypobranchial plate with notable lateral hypobranchial process, tip sub-truncated. Hypobranchial process at level of the second (CBII) and third (CBIII) ceratobranchial. At level of CBII, *Commissura proximalis* I and II (CP) joined by a small process arised from hypobranchial I and by the lateral hypobranchial process. *Commissura proximalis* III absent. Branchial basket with four curved ceratobranchials and numerous lateral projections. Ceratobranchial I continuous with the hypobranchial plate by mean of the hypobranchial I, and it bears a notable, medially curved branchial process. Branchial process tip rounded. Spicula I–III long, curved postero-dorsally; spicula IV shorter, straight (Fig 16C). Second and third ceratobranchials with free branchial process, notable on CBIII, not fused between each. Ceratobranchials distally joined by terminal commissures. Posterior process of ceratohyal not connected to the anterior margin of the hypobranchial plate (Fig 16C).

#### Comparisons

The tadpoles of *Ikakogi ispacue* sp. nov. differ from others of the family in their slightly pigmented dorsal coloration in preservative (strongly pigmented in *Vitreorana helenae*, *Sachatamia albomaculata*, *S*. *ilex*, *Teratohyla spinosa* and *T*. *pulverata*; Table 5). *Ikakogi ispacue* sp. nov. has a distinctively arch-shaped upper jaw sheath, whereas the the upper jaw sheaths are M-shaped in *Espadarana andina*, *Cochranella euknemos*, *Co*. *granulosa*, *Hyalinobatrachium aureoguttatum*, *H*. *orientale*, *H*. *taylori*, *H*. *valeroi* and *Nymphargus grandisonae* (Table 5). The spiracle is closer to the posterior margin of the body in *I*. *ispacue* sp. nov. (84.1% of BL), whereas it is approximately midway between the snout and the posterior margin of the body in *Hyalinobatrachium ibama* (stages 31and 36; 59.0 and 57.0% of BL, respectively). In lateral view the snout is acuminate in contrast to the rounded snouts of *Centrolene hesperium*, *Celsiella revocata*, *Cel*. *vozmedianoi*, *Cochranella granulosa*, *Co*. *resplendens*, *Hyalinobatrachium aureoguttatum*, *H*. *chirripoi*, *H*. *colymbiphyllum*, *H*. *duranti*, *H*. *fleischmanni*, *H*. *ibama*, *H*. *orientale*, *H*. *taylori*, *H*. *valerioi*, *H*. *vireovittatum*, *N*. *grandisonae*, *Vitreorana castroviejoi* and *V*. *helenae* (Table 5). The labial tooth row formula 2(2)/3 distinguishes *I*. *ispacue* sp. nov. from *Centrolene altitudinale* (LTRF: 1/2-3), *Ce*. *savagei* (LTRF: 2(1)/3 and 1(1)/2(2)), *Ce*. *daidaleum* (LTRF: 1/2 and 2(2)/2), *Co*. *guayasamini* (LTFR: 1/3), *H*. *cappellei* (LTRF: 0/2 and 0/3), *V*. *eurygnatha* (LTRF: 0/0) and *V*. *uranoscopa* (LTRF: 1/1 to 2/2; Table 5). The tadpole of *I*. *ispacue* sp. nov. is almost indistinguishable from the larvae of *I*. *tayrona*, however they differ in their internal morphology, specifically in the presence of 13 lateral buccal floor papillae in the larvae of the *I*. *ispacue* sp. nov. (Fig 11B), 10 lateral buccal floor papillae in *I*. *tayrona* sp. nov. (Fig 20A). Additionally, both species have five conical papillae in the buccal roof arena, whereas in the larva of *I*. *ispacue* sp. nov. three of them are lateral and two are medial, Fig 11B (all the papillae are laterally located in *I*. *tayrona*, Fig 20B) and the basihyal is present in the larvae of *Ikakogi tayrona* (n = 2) and absent in the larvae of *I*. *ispacue* sp. nov. (n = 2; Fig 16). However, given the small sample studied by us, the occurrence of the basihyal should be interpreted with caution (see Discussion section ahead).

### Redescription of the tadpole of *I*. *tayrona* (Ruiz-Carranza and Lynch, 1991b)

The tadpole of *Ikakogi tayrona* has been mentioned in two articles. [41] presented a brief description of a series of specimens identified as *Geobatrachus walkeri*, a species with direct development found in the Cuchilla of San Lorenzo, Santa Marta area in Colombia, and in [49], which cited few external larvae features (i.e. LTRF, spiracle position, snout shape and body coloration). Despite being very valuable, these descriptions lack complete illustrations and were based on a single or small series not taking into account intraspecific variation, thus limiting several characteristics necessary for its identification and species comparisons.

#### External morphology

**Fig 17:**
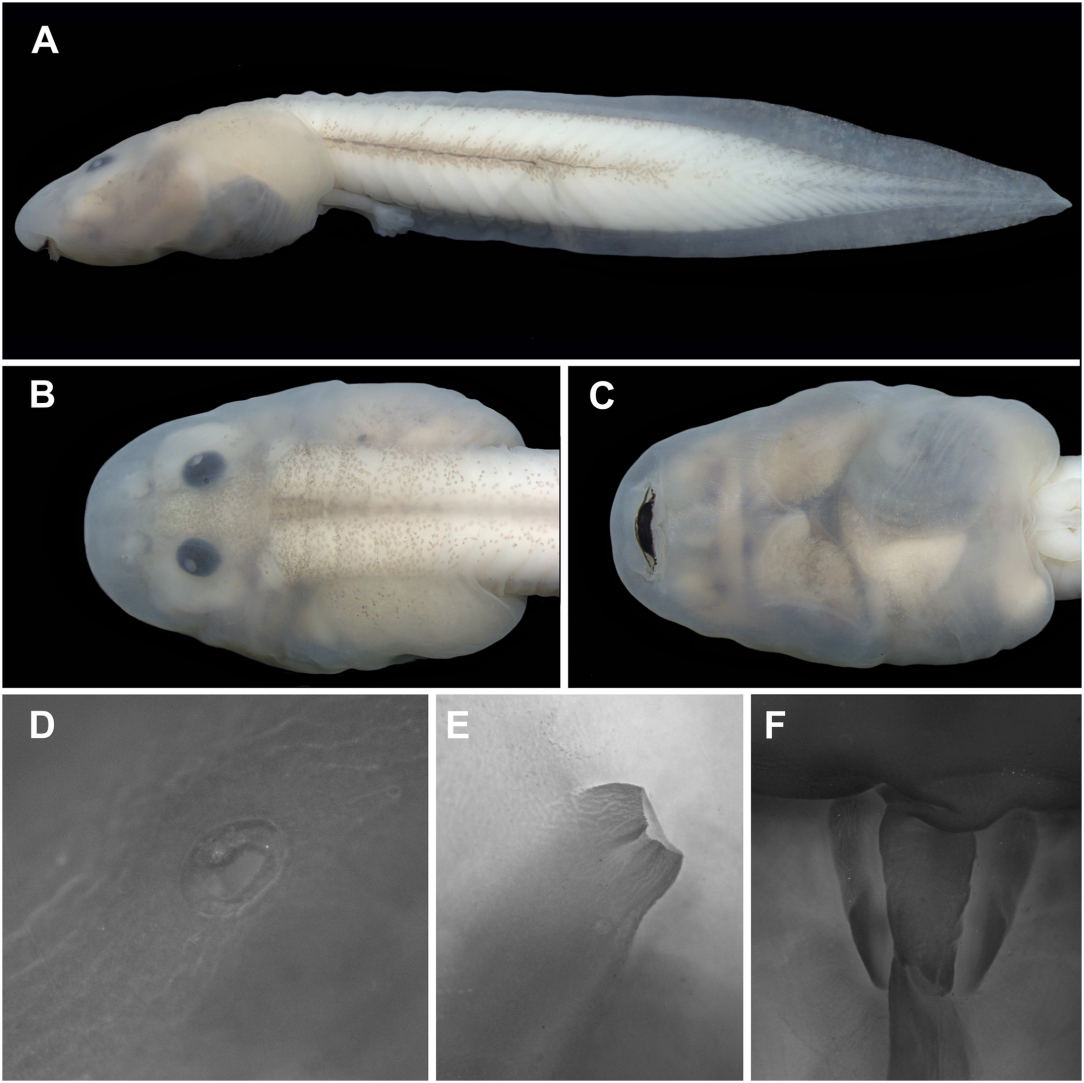
Tadpole in stage 35 of *Ikakogi tayrona* in lateral view. (A) dorsal and ventral views (B–C). Note the short spiracle and the translucent venter (C), nostril, spiracle and vent tube respectively (D–F). CBUMAG: ANF 01017 stage 35. Scale bar equal to 2 mm (A–C).

**Fig 18:**
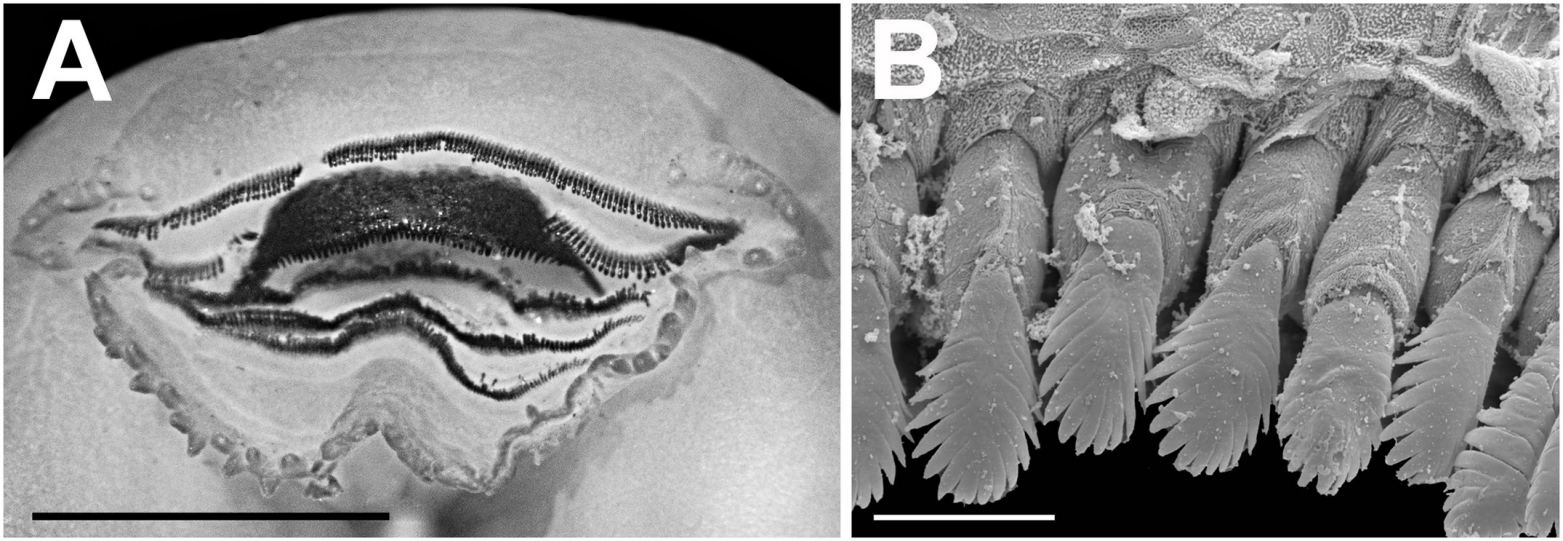
Oral disc of the tadpole of *Ikakogi tayrona*. (A) and lateral teeth of upper jaw; scale bar equal to 1.5 mm. (B), CBUMAG: ANF 01017, stage 35. Scale bar equal to 20 μm.

External morphology characters and general morphology of the tadpoles of *Ikakogi tayrona* are identical to those of *Ikakogi ispacue* sp. nov (Figs 17 and 18; n = 1 at Gosner’s stage 35, CBUMAG: ANF 01017; Tables 5 and 6).

**Table 6:** Mean and standard deviation of the morphological measurements (mm) of *Ikakogi tayrona* tadpoles.

Stage	N	TL	BL	BW	BH	IND	IOD	SSD	ESD	MTH	TAL	TMH	ODW	VTL
24	9	23,0 ± 1,2	7,0 ± 0,5	4,0 ± 0,5	2,4 ± 0,5	0,8 ± 0,0	1,3 ± 0,2	5,8 ± 0,3	2,4 ± 0,3	3,2 ± 0,4	15,9 ± 0,9	2,0 ± 0,4	2,2 ± 0,2	0,7 ± 0,2
		(21,2–24,9)	(6,0–7,7)	(3,5–5,1)	(1,5–3,0)	(0,8–0,9)	(1,1–1,5)	(5,5–6,3)	(1,8–2,8)	(2,5–3,9)	(14,9–17,5)	(1,5–2,6)	(1,9–2,3)	(0,4–1,0)
25	12	27,0 ± 1,7	8,3 ± 0,4	4,5± 0,4	2,7 ± 0,4	1,4 ± 0,3	1,3 ± 0,2	6,6 ± 0,5	2,9 ± 0,4	3,7 ± 0,6	18,7 ± 1,5	2,2 ± 0,3	2,4 ± 0,2	0,9 ± 0,1
		(24,7–30,0)	(7,5–8,9)	(3,6–4,9)	(2,2–3,5)	(0,9–1,9)	(1,0–1,6)	(5,4–7,4)	(2,2–3,3)	(2,4–4,9)	(16,9–21,6)	(1,9–2,7)	(2,1–2,6)	(0,7–1,1)
26	14	28,2 ± 2,4	8,5 ± 0,8	4,6 ± 0,4	2,4 ± 0,4	1,5 ± 0,2	1,3 ± 0,2	6,8 ± 0,9	3,0 ± 0,5	4,3 ± 0,4	19,7± 1,8	2,3 ± 0,4	2,4 ± 0,2	0,9± 0,3
		(23,6–32,1)	(7,3–9,6)	(4,0–5,4)	(1,8–3,1)	(1,1–1,9)	(1,0–1,7)	(5,6–8,3)	(2,2–4,0)	(3,7–4,9)	(16,3–22,7)	(1,6–2,9)	(2,1–2,7)	(0,6–1,8)
27	8	29,8 ± 4,6	8,9 ± 1,6	4,9 ± 0,7	2,6 ± 0,7	1,4 ± 0,2	1,3 ± 0,4	7,3 ± 1,3	3,3 ± 0,8	4,6 ± 0,8	20,9 ± 3,2	2,5 ± 0,6	2,6 ± 0,4	0,9 ± 0,2
		(25,2–39,2)	(7,4–11,9)	(4,2–6,2)	(1,5–3,6)	(1,1–1,7)	(1,0–2,3)	(6,0–9,9)	(2,2–4,4)	(3,7–5,8)	(17,3–27,3)	(2,9–4,0)	(2,2–3,0)	(0,8–1,3
28	11	30,4 ± 2,2	9,0 ± 0,6	4,8 ± 0,3	2,3 ± 0,3	1,5 ± 0,2	1,2 ± 0,1	7,2± 0,8	3,2 ± 0,3	4,6 ± 0,4	21,4 ± 1,8	2,5± 0,2	2,6 ± 0,1	0,9 ± 0,1
		(26,8–35,2)	(8,2–10,1)	(4,5–5,5)	(2,0–2,3)	(1,3–1,8)	(1,1–1,4)	(4,9–8,0)	(2,8–3,5)	(3,9–5,3)	(18,4–25,1)	(2,1–2,8)	(2,4–2,8)	(0,7–1,0)
29	5	34,8 ± 2,6	10,8± 0,8	5,9 ± 0,6	3,4 ± 0,8	1,5 ± 0,3	1,4 ± 0,1	8,6 ± 1,1	4,1 ± 0,6	4,6 ± 0,6	24,1 ± 2,2	3,2 ± 0,3	2,9 ± 0,3	1,4 ± 0,3
		(32,6–39,0)	(9,8–12,0)	(5,3–6,7)	(2,6–4,7)	(1,1–1,8)	(1,2–1,5)	(7,3–10,4)	(3,6–5,0)	(3,9–5,2)	(22,1–27,0)	(2,8–3,6)	(2,6–3,6)	(0,9–1,7)
30	5	35,0 ± 1,5	10,3 ± 1,0	5,6 ± 0,5	2,6 ± 0,1	1,7 ± 0,2	1,4 ± 0,0	7,9 ± 2,7	3,7 ± 0,5	4,4 ± 0,7	24,7 ± 1,0	2,8± 0,2	2,5 ± 0,4	1,2 ± 0,1
		(32,8–37,0)	(9,2–11,4)	(5,1–6,2)	(2,5–2,8)	(1,5–1,9)	(1,3–1,4)	(4,3–11,6)	(3,2–4,5)	(3,6–5,2)	(23,6–25,9)	(2,6–3,1)	(2,0–3,0)	(1,0–1,3)
31	2	39,6 ± 4,4	11,2 ± 1,8	6,6 ± 0,3	3,5 ± 1,4	1,5 ± 0,5	1,8 ± 0,1	8,8 ± 1,9	4,3 ± 0,8	5,0 ± 0,6	28,4 ± 2,7	3,4 ± 0,5	3,1 ± 1,1	1,3 ± 0,2
		(36,5–42,7)	(9,9–12,4)	(6,5–6,8)	(2,5–4,5)	(1,2–1,8)	(1,7–1,8)	(7,5–10,1)	(3,8–4,9)	(4,5–5,4)	(26,6–30,3)	(3,1–3,7)	(2,4–3,9)	(1,1–1,4)
33	1	42,8	11,9	6,8	4,6	2,0	2,2	9,7	4,7	5,7	30,9	3,8	3,8	1,5
34	1	37,7	11,5	6,5	3,6	1,6	1,3	7,8	3,8	5,1	26,2	2,5	2,8	1,5
35	3	44,5 ± 5,8	12,1 ± 1,4	6,7 ± 0,8	4,2± 1,1	2,2 ± 0,3	1,9 ± 0,5	9,8 ± 2,9	4,6 ± 0,6	6,2 ± 1,4	32,4 ± 4,4	3,7 ± 0,4	3,2 ± 0,8	1,7 ± 0,3
		(38,1–49,3)	(10,5–13,0)	(5,8–7,5)	(4,7–3,0)	(1,9–2,5)	(1,6–2,4)	(7,1–12,9)	(3,9–5,0)	(4,6–7,0)	(27,7–36,3)	(3,3–4,0)	(2,3–3,8)	(1,4–1,9)
36	4	39,7 ± 4,8	11,7 ± 0,7	6,8 ± 0,8	3,7 ± 0,5	2,2 ± 0,3	2,2 ± 0,2	8,2 ± 2,0	4,2 ± 0,1	5,8 ± 1,1	28,0 ± 4,8	3,5 ± 0,7	2,9 ± 0,7	0,8 ± 0,7
		(35,5–46,0)	(10,7–12,2)	(5,7–7,5)	(3,0–4,2)	(1,9–2,5)	(1,8–2,2)	(6,0–10,6)	(4,0–4,3)	(5,0–7,3)	(23,5–33,8)	(3,1–4,5)	(2,3–3,6)	(0,8–1,5)
37	1	42,3	12,2	7,0	4,1	1,3	1,6	9,2	4,3	5,8	30,1	3,9	2,7	0,7
38	1	40,3	10,5	6,7	3,5	1,6	1,2	9,0	3,8	5,0	29,8	2,7	2,4	1,0
39	1	42,9	11,9	7,3	4,9	1,8	2,7	9,0	4,1	6,2	31,0	3,9	2,8	0,0
40	3	44,2 ± 3,2	12,1 ± 1,5	7,06 ± 0,9	3,3 ± 0,6	1,7 ± 0,3	2,2 ± 0,3	9,2 ± 0,5	4,2 ± 0,4	6,1 ± 0,9	32,1 ± 1,9	3,9 ± 0,2	2,8 ± 0,2	1,0 ± 0,3
		(40,8–46,9)	(10,7–13,7)	(6,2–7,9)	(2,6–3,7)	(1,5–1,9)	(1,9–2,5)	(8,7–9,6)	(3,8–4,6)	(5,3–6,8)	(30,0–32,3)	(3,8–4,1)	(2,5–2,9)	(0,7–1,2)
41	4	44,8 ± 1,8	12,9 ± 0,7	7,4 ± 0,3	3,1 ± 1,3	1,6 ± 0,5	2,5 ± 0,3	9,6 ± 0,1	4,1 ± 0,1	5,5 ± 0,2	31,9 ± 1,6	3,8 ± 0,2	2,8 ± 0,2	0,0
		(43,6–47,5)	(11,9–13,6)	(7,1–7,1)	(1,5–4,6)	(1,2–2,3)	(2,1–2,8)	(9,5–9,7)	(4,0–4,2)	(5,3–5,7)	(30,4–34,0)	(3,6–4,0)	(2,6–3,0)	0,0
43	1	42,2	14,2	6,1	4,3	1,3	4,4	5,1	3,0	4,8	28,0	3,3	2,8	0,0

Mean ± standard deviation; range into parenthesis. Meristic measurements were: (TL) total length; (BL) body length; (BW) body width; (BH) body height; (IND) internarial distance; (IOD) interorbital distance; (SSD) spiracle-snout distance; (ESD) eye-snout distance; (TAL) tail length; (ESD) eye-snout distance; (MTH) maximum tail height; (TMH) tail muscle height; (ODW) oral disc width; (VTL) vent tube length.

#### Measurements

TL = 46.0; BL = 12.8; BW = 6.8; BH = 4.7; DFH = 1.7; VFH = 1.6; ED = 0,9; IND = 2.2; IOD = 2.4; SSD = 9.8; ESD = 4.6; MTH = 6.8; TMH = 3.9; TMW = 3.0; TAL = 33.2; ODW = 3.8; VTL = 1.8.

#### Color in life

**Fig 19:**
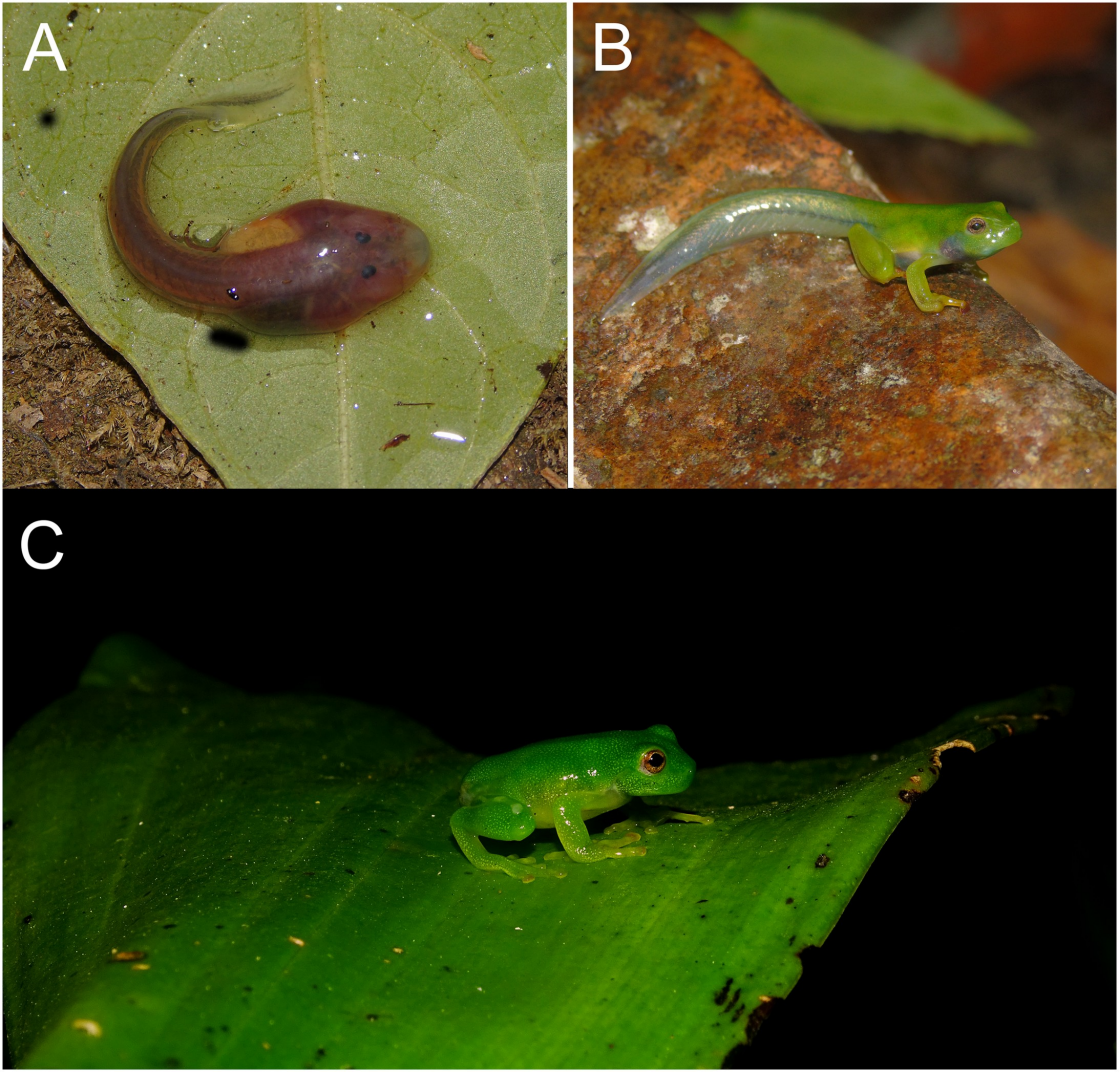
Live specimens of *Ikakogi tayrona* tadpole near San Lorenzo, Gaira River, SNSM. Note the red color due of hypervascularization of skin (A), green froglet (B) and postmetamorphic, SVL = 13.9 mm (C). CBUMAG: ANF 00960. Photos A and B not to scale.

Highly vascularized skin, which gives the larvae a dark red or pink coloration (body and tail musculature). Dorsum slightly pigmented but with some minute scattered pigmentation especially concentrated between eyes, posterior section of body and on myotomes across tail. Ventral skin of body translucent, intestine translucent but with brown or dark green color because of the intestinal content, liver dark reddish, heart and other parts of the circulatory system red. Tail musculature red with conspicuous medial brownish longitudinal stripe in the midline of myotomes, extending from the tail-body junction until almost three half 1/3 of the tail. Tail fins are transparent but with minute melanophores mainly in distal surface (Fig 19).

#### Color in preservative

The color pattern is similar to that of living tadpoles, but loses its red or pink coloration. The dorsum, tail musculature and venter light cream. Melanophores in dorsum, tail fins and tail musculature turn pale gray (Fig 17A–17C).

#### Variation and ontogenetic development

Variation of 13 morphometric characters of tadpoles in Stages 24–43 are given in Table 6. Tadpoles of stages 24–25 invariably presented tooth rows slightly keratinized, poorly developed and/or in some areas interrupted. The labial tooth row formulae from stage 25 to 34 is (2(2)/3), and from stage 35 to 40 is 2(2)/2. In late stages (41–43) a gradual reduction of LTRF is also observed, changing from 1(1)/2; 2(2)/1; 1/1; 0/2 to 0/0.

In some specimens the middle dark brown band of the tail musculature are either interrupted or incomplete; its extension slight varies between the half and posterior third of the tail musculature. The tail tip is bluntly pointed (Fig 17A) or rounded. The eyes of *Ikakogi* are small, scarcely pigmented and C-shaped in dorsal view in stages 25 to 28, whereas they are bigger, pigmented and round when they reach the stage 35 and latter (Fig 17B). Major differences in pigment pattern were observed in the sample: tadpoles from the stage 41 to 43 showed a uniformly greenish color pattern in the dorsal skin and limbs and the eyes are oriented anterolaterally. Also, the snout was rounded or truncate in dorsal and lateral views (Fig 19A–19C).

#### Internal morphology

**Fig 20:**
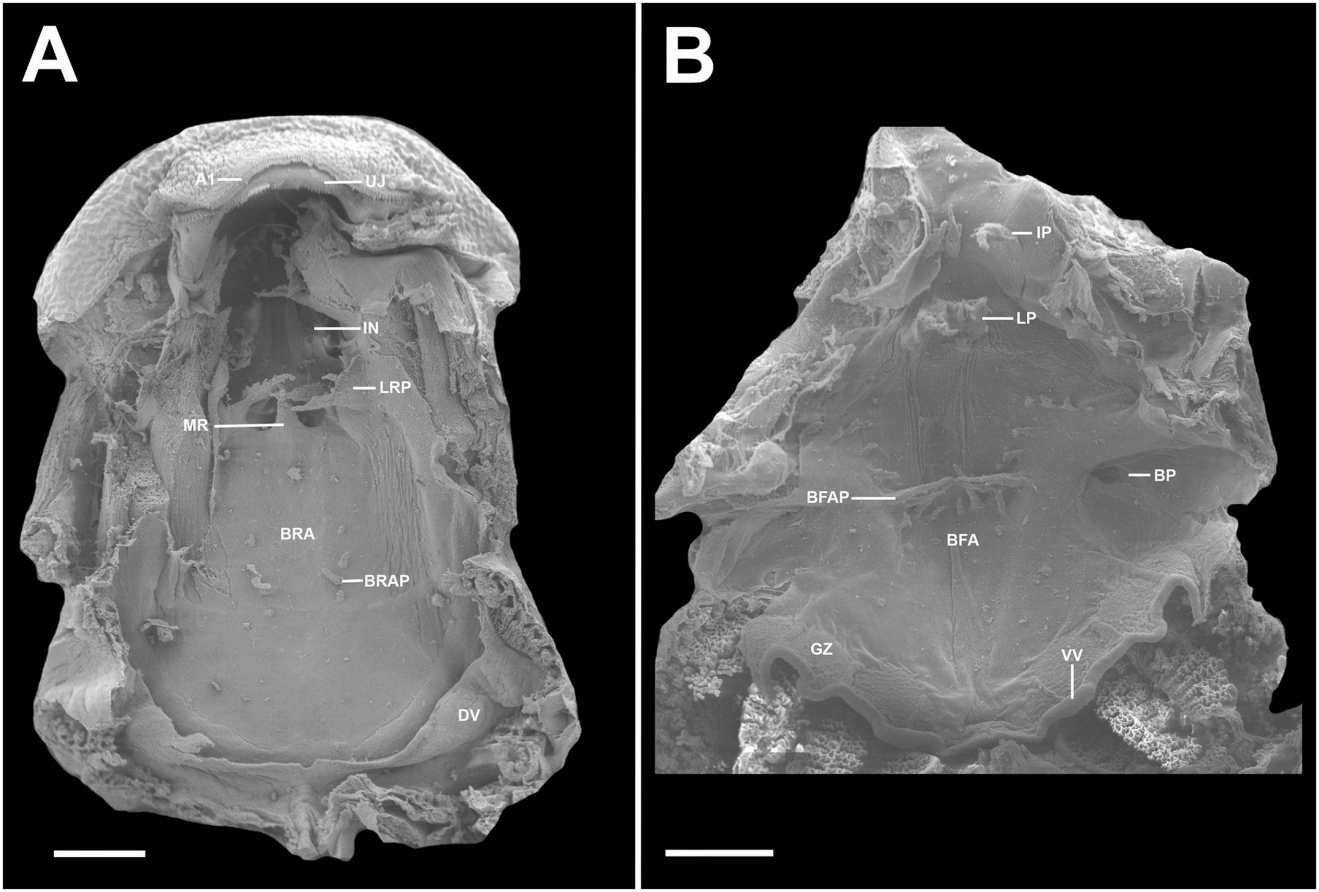
Buccopharyngeal morphology of the tadpole of *Ikakogi tayrona*. Buccal roof (A), and buccal floor (B). Abbreviations: A1, first anterior tooth row; BFA, buccal floor arena; BFAP, buccal floor arena papillae; BP, buccal pocket; BRA, buccal roof arena; BRAP, buccal roof arena papillae; DV, dorsal velum; GZ, glandular zone; IL, infralabial papillae; IN, internal nares; LP, lingual papillae; LRP, lateral ridge papillae; PNP, postnarial papillae; PP, prepocket papillae; UJ, upper jaw sheath; VV, ventral velum. ICN 58308, stage 29. Scale bar equal to 500 μm.

**Fig 21:**
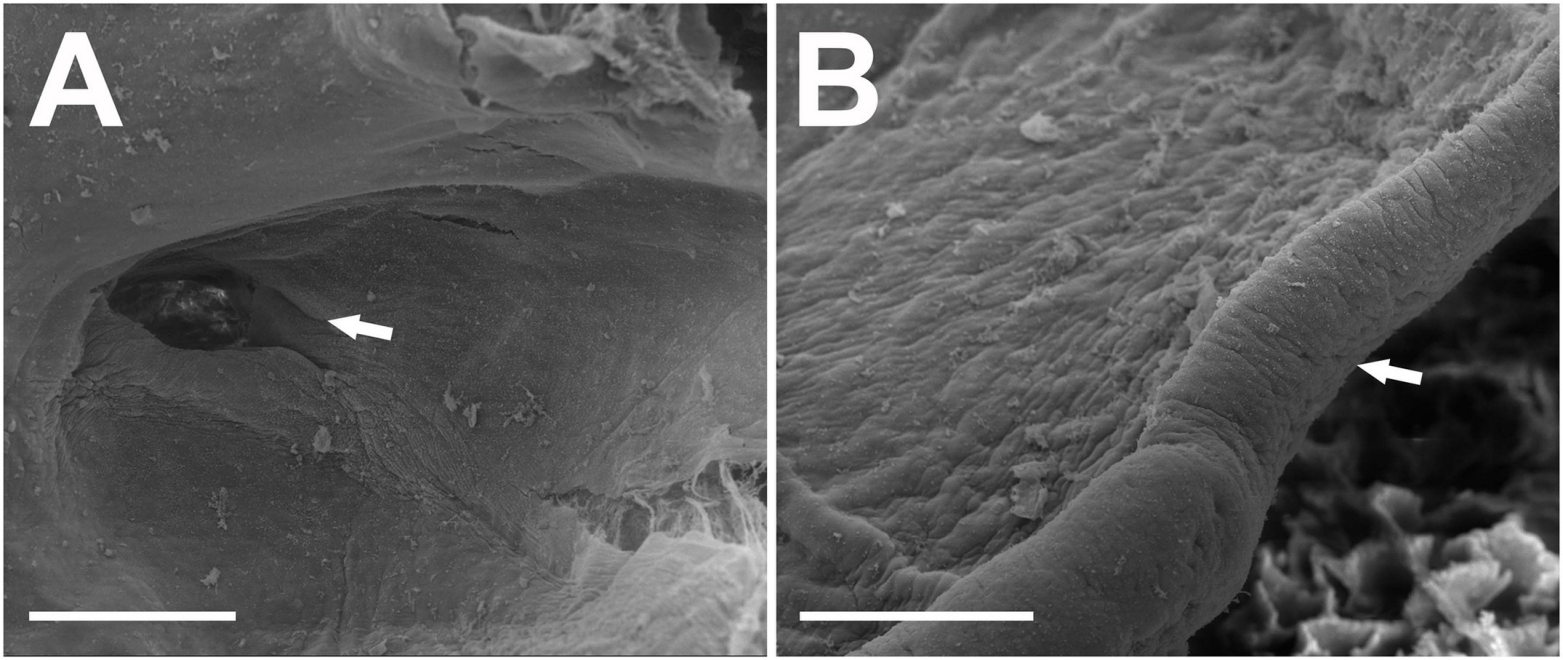
Detail of the concealed buccal pocket perforation in the buccal floor of *Ikakogi tayrona*. (A). Detail of pores on the ventral velum (B). ICN 58308, stage 29. Scale bar equal to 200 μm (A) and 100 μm (B).

**Buccopharyngeal cavity**. The characteristics of the buccal roof and floor are almost identical in *Ikakogi tayrona* and *I*. *ispacue* sp. nov. with the following exceptions: (1) five tall and conical papillae present in the buccal roof arena, all laterally located and, (2) 13 lateral buccal floor arena papillae in *I*. *tayrona* (Figs 20 and 21).

#### Larval muscles

**Fig 22:**
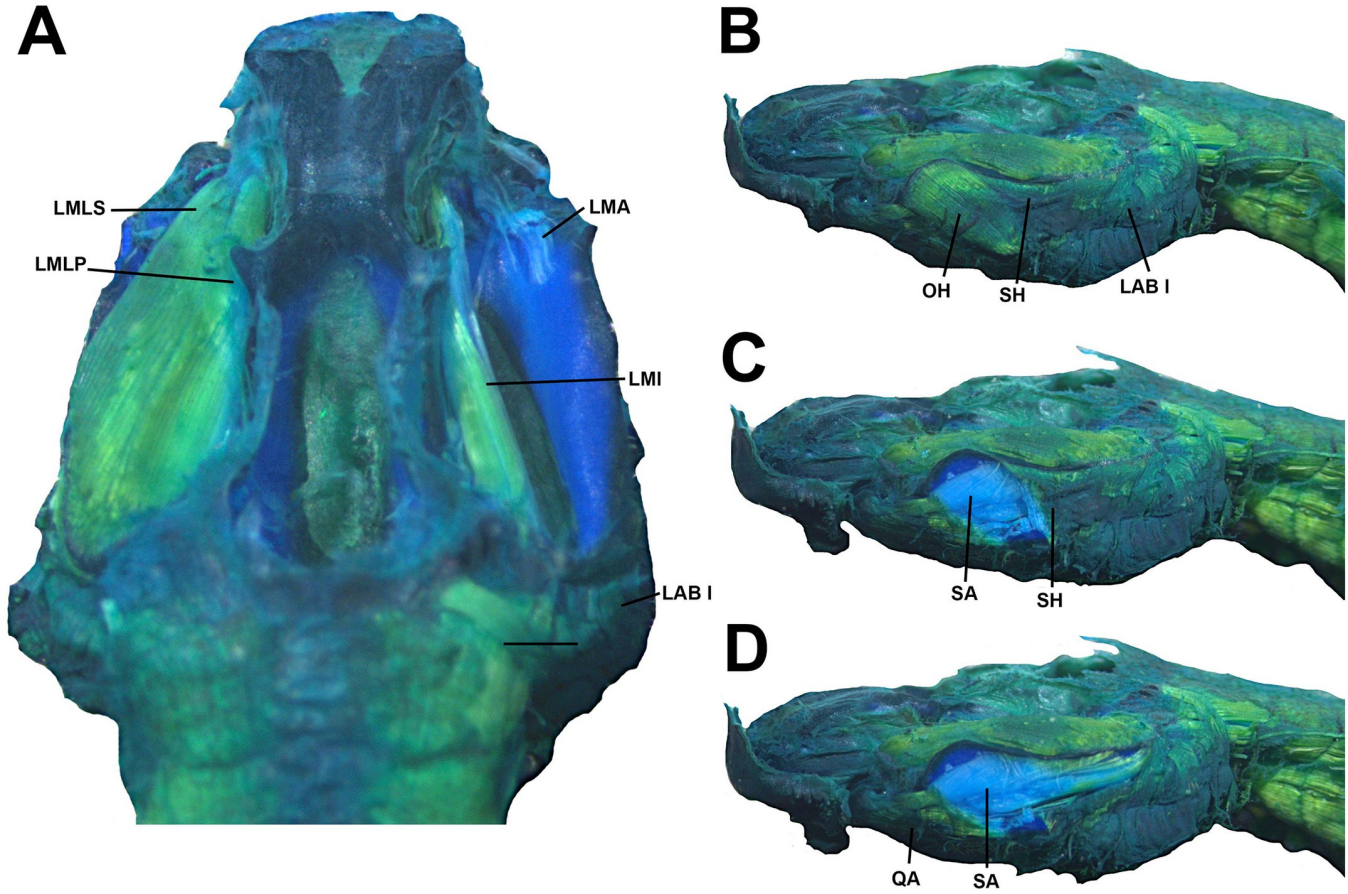
Cranial muscles of the tadpole of *Ikakogi tayrona*. Dorsal (A) and lateral views (B-D). Abbreviations: LAB (I to III), *musculus levator archus branchialum* I to III; LMA, *m*. *levator mandibulae articularis*; LMI, *m*. *levator mandibulae internus*; LMLP, *m*. *levator mandibulae longus profundus*; LMLS, *m*. *levator mandibulae longus superficialis*; OH, *m*. *orbitohyoideus*; QA, *m*. *quadratoangularis*; SA, *m*. *suspensorioangularis*; SH, *m*. *suspensoriohyoideus*. CBUMAG: ANF 01018 stage 35. Scale bar equal to 1.0 mm.

The origin and insertion of all muscles are identical in *I*. *tayrona* and *I*. *ispacue* sp. nov. (see S5 Appendix, Fig 22).

#### Sinus hyobranchialis

**Fig 23:**
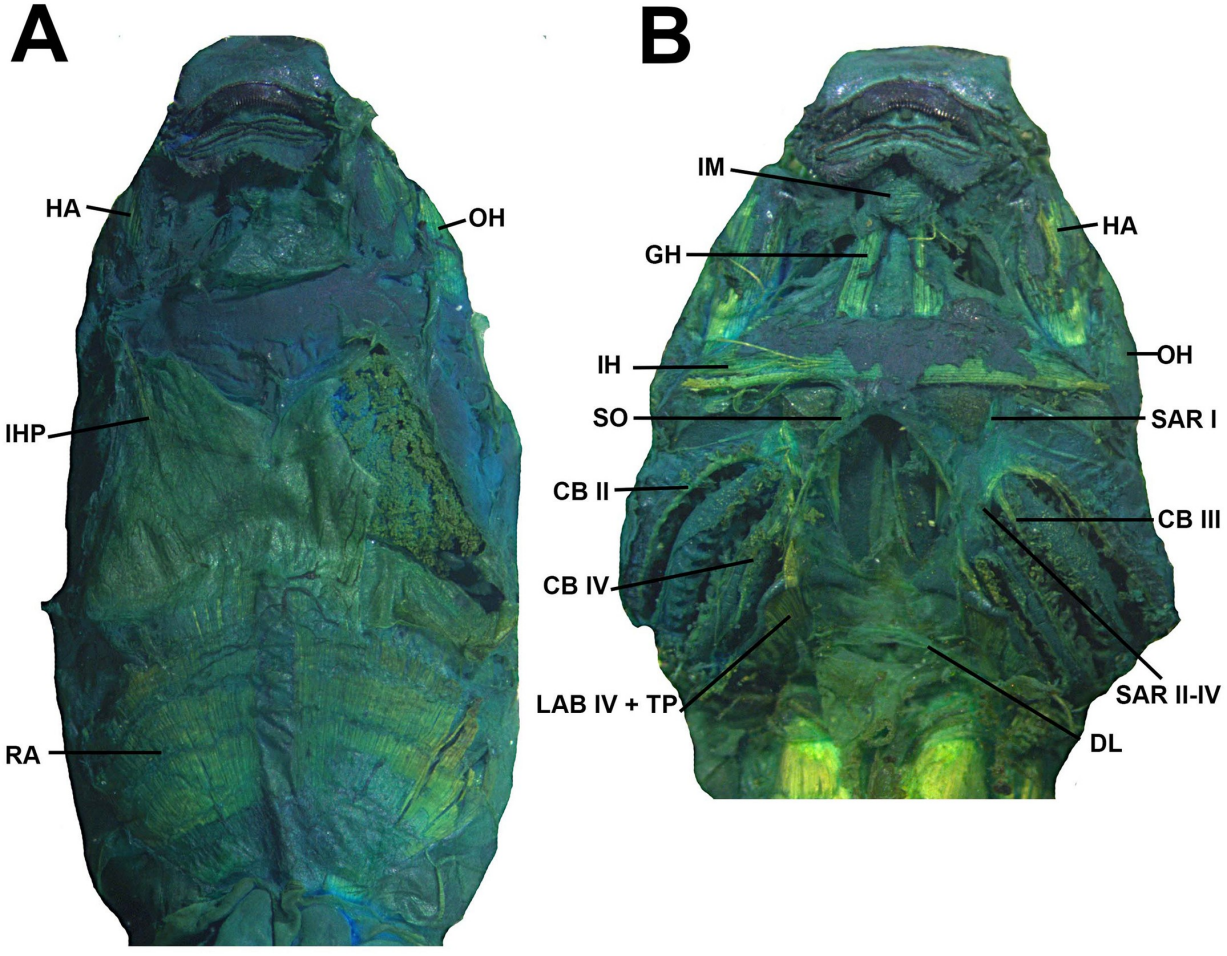
Cranial muscles of the tadpole of *Ikakogi tayrona* in ventral view (A–B). Abbreviations: CB (II-IV), *musculus constrictor branchialis* II to IV; GH, *m*. *geniohyoideus*; HA, *m*. *hyoangularis*; IH, *m*. *interhyoideus*; IHP, *m*. *interhyoideus posterior*; IM, *m*. *intermandibularis*; OH, *m*. *orbitohyoideus*; RA, *m*. *rectus abdominis*; RC, *m*. *rectus cervicis*; SAR I, *m*. *subarcualis rect*us I; SO, *m*. *subarcualis obliquus*. CBUMAG: ANF 01018 stage 35. Scale bar equal to 1.0 mm.

There are no differences between *Ikakogi tayrona* larvae and *I*. *ispacue* sp. nov (Fig 23).

#### Skeleton

**Fig 24:**
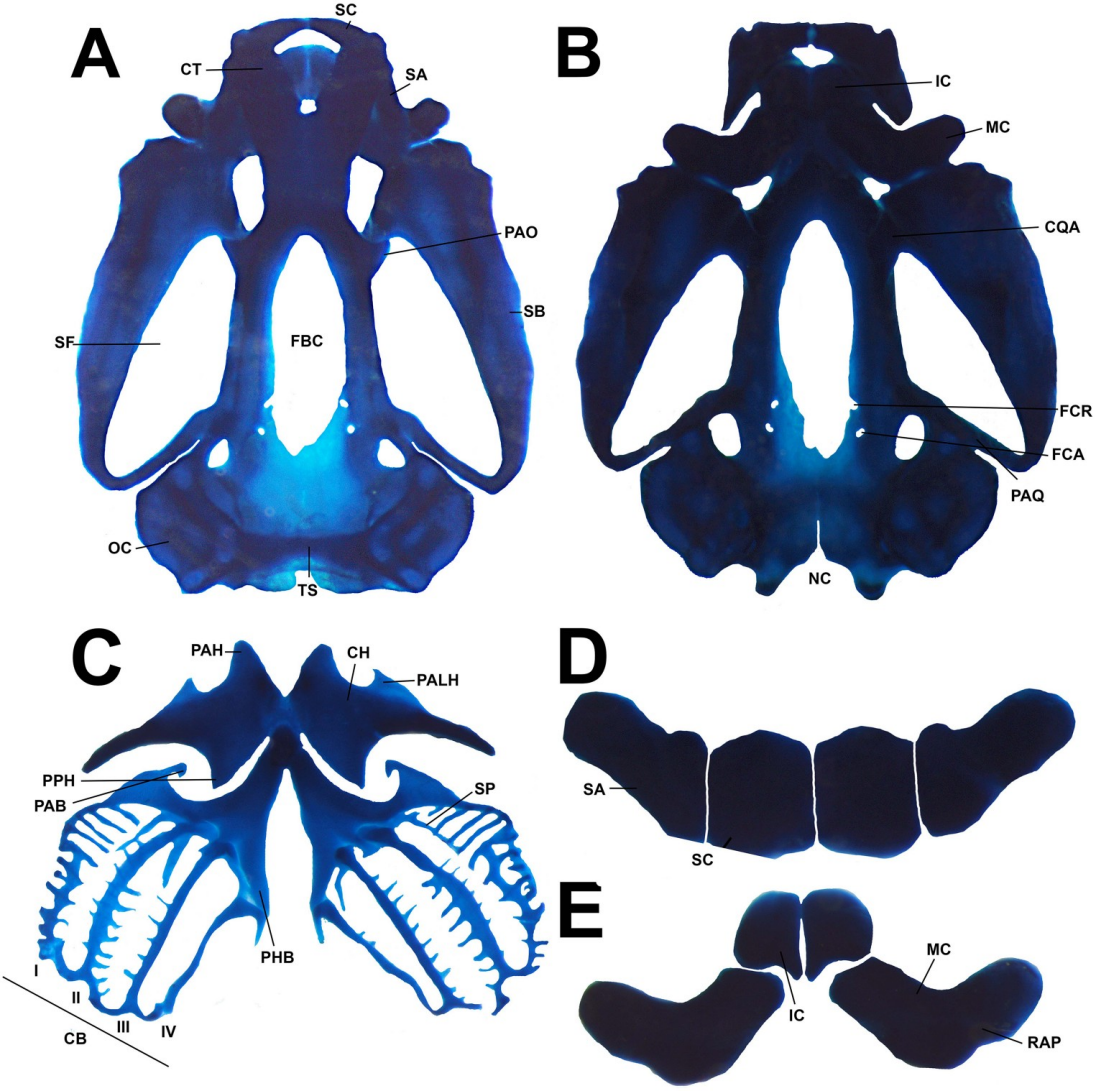
Larval chondrocranium morphology of the tadpole of *Ikakogi tayrona*. Dorsal (A), and ventral views (B), *apparatus hyobranchial* (C), suprarostral (D) and infrarostral cartilage with Meckel´s cartilage (E). CBUMAG: ANF 01018 stage 35. Abbreviations: BH, basihyal; CB (I-IV), ceratobranchials I to IV; CH, ceratohyal; CQA, *commissura quadratocranialis*; CT, *cornua trabeculae*; FBC, *fenestrae basicranialis*; FCA, *foramen caroticum primarum*; FCR, *foramen craniopalatinum*; IC, infrarostral cartilage; Meckel`s cartilage; NC, notochord canal; PAB, *processus anterior branchialis*; PAH, *processus anterior hyalis*; PALH, *processus anterolateralis hyalis*; PAO, *processus antorbitalis*; PHB, *planum hypobranchialis*; PPH, *processus posterior hyalis*; RAP, retroarticular process of Meckel`s cartilage; SA, suprarostral ala; SB, subocular bar; SC, suprarostral corpora; SF, *subocular fenestrae*; SP, spicules. Scale bar equal to 1.0 mm.

The internal morphology of *Ikakogi tayrona* larvae is similar to the descriptions provided before for *I*. *ispacue* sp. nov. with the following exceptions: (1) the anterior margin of trabercular horns is rounded without medial projection; (2) the oculomotor foramen is closed totally; (3) the small ovoid *corpus* cartilage between ceratobranquial I and palatoquedrate is present; (4) the Basihyal (copula I) is absent; (5) in one tadpole in stage 35, the right CBIII is joined to the *planun hypobranchiale* by a weak and thin cartilage at level of its spicula and, (6) in stage 35, the posterior process of ceratohyal is joined by a connective tissue to the anterior margin of the hypobranchial plate (Fig 24).

#### Comparisons

The tadpole of *Ikakogi tayrona* differs from those of other centrolenids by its slightly pigmented dorsum coloration in preservative (strongly pigmented in *Sachatamia albomaculata*, *S*. *ilex*, *Teratohyla spinosa*, *T*. *pulverata* and *Vitreorana helenae*; Table 5). In lateral view the snout of the tadpole of *I*. *tayrona* is acuminated unlike a rounded snout that is found in *Centrolene hesperium*, *Celsiella revocata*, *Cel*. *vozmedianoi*, *Cochranella granulosa*, *Co*. *resplendens*, *Hyalinobatrachium aureoguttatum*, *H*. *chirripoi*, *H*. *colymbiphyllum*, *H*. *duranti*, *H*. *fleischmanni*, *H*. *ibama*, *H*. *orientale*, *H*. *taylori*, *H*. *valerioi*, *H*. *vireovittatum*, *N*. *grandisonae*, *Vitreorana castroviejoi* and *V*. *helenae* (Table 5). *Ikakogi tayrona* has an arch-shaped upper jaw sheath, whereas the compilation of the upper jaw sheaths in Centrolenids describe the sheath to be M-shaped for *Espadarana andina*, *Co*. *euknemos*, *Co*. *granulosa*, *Hyalinobatrachium aureoguttatum*, *H*. *orientale*, *H*. *taylori*, *H*. *valeroi* and *N*. *grandisonae*. The labial tooth formula 2(2)/3 distinguishes *I*. *tayrona* from *Centrolene altitudinale* (LTRF: 1/2-3), *Ce*. *daidaleum* (LTRF: 1/2 and 2(2)/2), *Ce*. *savagei* (LTRF: 2(1)/3 and 1(1)/2(2)), *Co*. *guayasamini* (LTFR: 1/3), *H*. *cappellei* (LTRF: 0/2 and 0/3), *V*. *eurygnatha* (LTRF: 0/0) and *V*. *uranoscopa* (LTRF: 1/1 to 2/2), Table 5. Externally, the tadpole of *I*. *tayrona* is indistinguishable from the larvae of *I*. *ispacue* sp. nov., however they can be differentiated by their internal morphology, specifically, by the presence of ten lateral buccal floor papillae in the larvae of the *I*. *tayrona* (Fig 24B), 13 lateral buccal floor papillae in *I*. *ispacue* sp. nov. (Fig 11B). They can also be differentiated because the larva of *I*. *tayrona* have five conical papillae located laterally in the buccal roof arena (Fig 24A), whereas the papillae are located laterally (three) and medially (two) in *I*. *ispacue* sp. nov. (Fig 11A), and because the basihyal is absent in the larvae of *Ikakogi ispacue* sp. nov., present in *I*. *tayrona* (Fig 24), but see Discussion section below.

#### Oviposition site, egg clutches, and tadpole habitat

Females deposited and cared for egg clutches (referred to the species by association with parental perched on clutches) on the upper or lower side of leaves overhanging streams (ca. 0.5–2 m). Clutches of *Ikakogi tayrona* consisted of 53 cream or very pale green eggs (n = 6; ±10.21; 40–71 eggs); the mean size of eggs in clutches varies from 3.2 to 5.7 mm (see Table 7). The morphology of the egg mass observed near males of *I*. *tayrona* is a monolayer mass usually lacking eggs and jelly in the center of the clutch, which gives an appearance of a "ring" shape (n = 6). Embryos exhibit a hyper-vascularization of head, resulting in a reddish or pink head; the heart is translucent but reddish by blood. The tadpoles of *Ikakogi tayrona* were collected buried in sand in small backwaters and ponds at the border of streams. The area of the ponds was 3–4 m^2^, with a maximum depth of 50–80 cm.

**Table 7:** Features of egg clutches of the glassfrog *Ikakogi tayrona* in a population at Serrania de San Lorenzo, Sierra Nevada de Santa Marta, Colombia.

Developmental stage (according to [83])	Number of eggs	Egg size (mm, mean±SD)	Embryo color
9–13	52	3.0±0.30 (2.0–2.7)	white
9–13	71	3.4±0.58 (2.2–4.6)	white
17–19	53	4.4±0.56 (3.2–5.3)	whitish
19–21	50	4.8±0.62 (3.7–6.2)	yellowish
22–23	40	4.8±0.37 (4.0–5.6)	yellowish body; pink head; belly and heart reddish
24–25	58	6.6±0.68 (5.4–7.8)	yellowish body; pink head pink; belly and heart reddish

## Discussion

Anurans of the family Centrolenidae are a diverse clade of arboreal frogs distributed across the Neotropical region [24]. As currently known and including the species here in described, Centrolenidae contains 158 species, 50% of which occur in Colombia [24]. Recently, several species of centrolenids have been described or documented to occur in that country, and more are expected to be discovered as research continues in biologically unexplored and topographically complex regions (e.g. [12, 15, 16]).

The genus *Ikakogi* was extracted from *Centrolene sensu lato*, which was found to be non-monophyletic [5]. *Ikakogi tayrona* and its putative sister species *I*. *ispacue* sp. nov. are medium-sized centrolenid frogs that are endemic to the Sierra Nevada of Santa Marta [70]. Males of both species emit calls from the upper and lower surfaces of leaves and contrary to most centrolenid frogs, egg care is performed by females rather than males ([6, 80], present study).

Other recent studies have demonstrated that the diversity of the highly endemic herpetofauna of SNSM is underestimated (e.g. [84, 85, 81, 86]). These findings, coupled with the present study, suggest that the diversity of amphibians and reptiles in the SNSM of Colombia is greater than the current estimates and underscore the need to conduct additional surveys of the SNSM fauna.

### Comments on the systematics of *Ikakogi*

Recent molecular-based phylogenetic analyses have greatly improved centrolenid systematics [5, 7, 10, 11]. As a result of these advances, Guayasamin et al. [5] proposed a revised taxonomy composed of 12 genera. However, some disagreement remains regarding the relationships among some genera, especially within the tribe Cochranellini, but also regarding the phylogenetic position of *Ikakogi* [5, 11]. These disagreements are partly attributable to the conflicting phylogenies among different studies that used dissimilar aligment and optimality criteria (e.g. [5, 10, 11]).

Previous studies found *Ikakogi* to be the sister of all other glassfrogs [5, 7, 10, 11] or sister taxon of the subfamily Centroleninae [8]. The external morphology of *Ikakogi* is similar to that of species of *Centrolene* and *Espadarana* (i.e., they share some character states like humerus with a conspicuous spine and translucent peritonea); however, they seem to be most similar to species of Hyalinobatrachinae. For example, some morphological traits of adults, such as dorsal coloration in preservative (cream to very pale lavender), bone coloration (white or very pale green concentrated in bone ephiphyses), tympanic membrane and annulus conspicousness (hidden), egg clutch location (under/upper side of leaves), egg clutch (monolayer), and oocyte pigmentation (cream to very pale green) are intriguing and shared mainly with species of *Hyalinobatrachium* and *Celsiella*. Because *Ikakogi tayrona* is considered the sister taxon of all other centrolenids (but with some remaining phylogenetic problems; see 5, 7, 8, 11), our records will offer insights into the ancestral states for egg attendance, egg clutches arrangement and location, and tadpole morphology. Future work incorporating all available molecular and morphological evidence in a phylogenetic analysis is necessary to improve our appreciation of relationships of *Ikakogi* genus and centrolenids and will further our understanding of the origin and evolution of their morphological characters.

### Evidence from vocalizations, egg clutches and larval morphology

*Ikakogi tayrona* and *I*. *ispacue* sp. nov. provide a clear illustration of the relevance of studying multiple sources of evidence to test taxonomic hypotheses. The two species are identical in all adult external morphological characteristics usually used to identify species of centrolenids. In contrast, comparison of vocalizations, DNA sequences, and internal larval morphology (including the number of lateral buccal floor papillae, the spatial arrangement of the five conical buccal roof papillae, and the basihyal structure in the condochranium) revealed diagnostic differences that support the hypothesis that the two groups are heterospecific.

The description of centrolenid advertisement calls facilitates species-level taxonomy and provides phylogenetically and ecologically relevant information [87]. The advertisement calls of *Ikakogi tayrona* and *I*. *ispacue* sp. nov. have several remarkable differences. For example, in addition to differences in dominant frequency, calls are shorter and comprise multi-pulsed notes in *I*. *ispacue* sp. nov., whereas they are longer and non-pulsed in *I*. *tayrona* (see Table 2, and [80]).

Reproductive behavior, natural history observations and tadpole morphology are important for making appropriate decisions in taxonomy, but they are also useful to test evolutionary hypotheses about the diversification of glassfrogs specifically and anurans generally (e.g. [88–91] and [86]). Glass frogs have a well-established reproductive mode consisting of the adherence of egg clutches on substrates overhanging water [92, 93]. Detailed quantitative data for most of these traits are absent for most species [9, 17, 18, 94–99]. Likewise, centrolenid tadpoles are poorly known and to date they have been formally described for less than 40 species of the 158 species known diversity (e.g. [100, 101, 49–53]).

### Buccopharyngeal anatomy

Data on centrolenid buccopharyngeal anatomy is extremely restricted, available only for *Hyalinobatrachium fleischmanni* [64]. Comparison of our data with those of Wassersug [64] shows that both *Ikakogi* and *Hyalinobatrachium* share some character states, such as the elongate buccal floor and roof, two pairs of infralabial papillae (one pair adjacent to the midline and the other placed over Meckel’s cartilage), four lingual papillae, few papillae and pustulations in the buccopharyngeal cavity, longitudinally oriented internal nares with vacuities, tall conical median ridge, and slit-like buccal pocket. Given that these two genera do not appear to be closely related ([5, 8, 10, 11] but see comments on the systematics of *Ikakogi* above), the shared occurrence of those states may represent putative synapomorphies for Centrolenidae. Nevertheless, *Ikakogi* present projections on the ventral vellum margin, papillae on the arena of the floor and roof, and more (three pairs) postnarial papillae, states absent in *Hyalinobatrachium fleischmanni*. Our results support the importance of the buccopharyngeal morphology for the systematics of glassfrogs, however, we stress the need for more species descriptions and data.

The elongate body observed in centrolenid larvae might create some difficulties in the interpretation of some characters. For instance, Wassersug [64] interpreted the large, tall, conical papillae near the median ridge as homologous to the lateral ridge papillae and questioned the homology of the “anterolateral compressed flap” papillae observed on each side of the buccal floor. In contrast we interpreted the large papillae near the median ridge as a third pair of postnarial papillae, which occupies this position (near the median ridge) as a consequence of the tadpole’s elongation. The lateral ridge papillae, in turn, are the features of unknown homology mentioned by Wassersug [64]. Our choice derives from the parsimony principle; in our hypothesis, rearrangements of some papillae may be the result of body’s elongation and the evocation of a new papilla is not needed to explain the observed configurations. We stress, however, that information from other species and developmental series is needed to better test the homology of these characters.

### Cranial muscles

Anuran cranial muscles are highly variable [62, 63, 102] and many different patterns of origin and insertion have evolved independently in several lineages. Frost et al. [2] based on Haas [63] data for *Cochranella granulosa*. Haas [63] included the *m*. *levator mandibulae externus* forming a single muscle and the *m*. *subarcualis rectus* II–IV having an anterior insertion on the *processus branchialis* III as candidate synapomorphies for Centrolenidae. Our data supports [2] hypothesis partially. We also found the *m*. *levator mandibulae externus* to be a single muscle; however, we observed the *subarcualis rectus* II–IV to reach the *processus branchialis* II, although some fibers also inserted anteriorly in the *processus branchialis* III. Given that one of the phylogenetic placements of *Ikakogi* is as a sister to all other centrolenids (the other one is sister of the subfamily Centroleninae, [8]), it is possible that the condition observed by us for *Ikakogi tayrona* and *Ikakogi ispacue* sp. nov. is plesiomorphic within Centrolenidae, with the shortening of this muscle being an evolutionary trend in the family.

Nevertheless, the most striking character of the cranial muscles of *Ikakogi* larvae is the origin of one slip of the *m*. *levator arcus branchialium* III on the dorsal surface of the otic capsule; not only is its origin peculiar, but so too is its trajectory until its insertion of the ceratobranchial III passing over the axial muscles (note that the axial musculature also extends further on the otic capsule). As far as we know, there is no record for such condition among anuran larvae; Haas [63] studied the cranial muscles of *Cochranella granulosa*, however he did not mention this character-state.

### Larval skeleton

Published information on the chondrocranium of Centrolenidae is limited to the character states of *Cochranella granulosa* by Haas [63]. Our observations on *Ikakogi* show that various character states are shared with *C*. *granulosa*; however, some differences were observed, such as the low suspensorium (intermediate in *C*. *granulosa*), the presence of the basihyal in *Ikakogi ispacue* sp. nov. (absent in *I*. *tayrona* and *C*. *granulosa*), the lack of a medial articulation between the hypobranchial plates (articulated in *C*. *granulosa*), and the presence of the *commissura proximalis* I and II (absent in *C*. *granulosa*).

Intraspecific variation in the basihyal has been described in dendrobatoids (see [60]) and interspecific variation in *Melanophryniscus* (Bufonidae, see [103]), dendrobatids [60] and Centrolenidae (this work). Although, the basihyal is absent in larvae of *Ikakogi tayrona* and present in *I*. *ispacue* sp. nov., given the small sample studied by us, the occurrence of the basihyal should be interpreted with caution. The phylogenetic value of the other differences found between *Ikakogi* and *Cochranella* have been showed in other frog families (e.g., [104, 105, 61, 63), which highlights the importance of increasing studies on the chondrocranium of centrolenids.

### Larval specializations for burrowing?

There are many records of fossorial habits in the literature for centrolenid larvae (e.g. [106, 38, 49, 54, 51, 52]). Altig and Johnston [107] placed centrolenids in the fossorial ecomorphological guild Type I and enumerated a series of characters for that guild: i) LTRF 2/3; ii) a small oral disc; iii) incomplete anterior marginal papillae; iv) very small eye (at least during the first stages but gradually increasing the size); and v) very often, red coloration due to highly vascular skin. These character-states are present in the larvae of several unrelated taxa such as *Leptobrachella* and *Leptolalax*: Megophryidae (e.g. [108–110]), *Micrixalus*: Micrixalidae [111], *Cardioglossa*: Arthroleptidae [112], *Otophryne*: Microhylidae [110], *Staurois*: Ranidae (e.g. [113]), and centrolenid larvae in general (e.g. [49, 51, 52]).

In addition to the characters proposed by [107], these larvae also present bodies that are compressed laterally and depressed dorsally, strong caudal muscles, shallow tail fins, and depressed and truncated snouts [49, 51]. Regarding the internal morphology of fossorial tadpoles, little is known. [110, 111] compared the musculoskeletal morphology of *Leptobrachella mjobergi* and *Otophryne robusta* and described the crania of *Micrixalus herrei*, [110] finding that these fossorial taxa differ greatly. Although the larvae of *Ikakogi* do not appear to share relevant characteristics with those of *O*. *robusta* [114], they do share several relevant characteristics with *M*. *herrei* and *L*. *mjobergi*, including long, laterally compressed crania, robust anterior cartilages (suprarostral and trabecular horns, Meckel’s cartilage), a low muscular process, a subocular bar narrowed posteriorly and a thin *processus ascendens*, and, in *Ikakogi* and *Leptobrachella*, weakly chondrified lateral cartilage of the *cavum cranii*, and thin ceratobranchials. The *m*. *levator mandibulae* group is massive and the paraxial musculature reaches far anterior on the otic capsule.

Lastly, we recorded the absence of lungs in both *Ikakogi* larvae. Several researchers (e.g. [115–117]) indicated the importance of the lungs for larval buoyancy. The absence of larval lungs, even in well-developed larvae (Gosner stages 30+) might be related to the fossorial habits. Regarding gas exchanging, the hypervascularization of the skin and branchial area, including the *sinus hyobranchialis*, probably compensate the absence of lungs and the failure to breath air. areal.

## Supporting information

S1 Appendix Specimens (tadpoles and call vouchers) of *Ikakogi tayrona* and *Ikakogi ispacue* sp. nov. (DOCX)

S2 Appendix Specimens (adults) used to comparisons and to reconstruct species distribution. A single asterisk (*) indicates no voucher specimens collected but species indentity inferred through examination of call by the authors. Double asterisk (**) denotes examination of photographs assigned to *Ikakogi* but with identity uncertain.
(DOCX)

S3 Appendix Uncorrected pairwise genetic distances (%) inferred from mitochondrial MT-Cytb2 gene. (DOCX)

S4 Appendix GenBank accession numbers for the Centrolenid species sequences employed in uncorrected pairwise genetic distances analysis. (DOCX)

S5 Appendix Cranial muscles codified for *Ikakogi tayrona* and *I*. *ispacue* sp. nov. (DOCX)
