# Supplementary material for: The poverty of adult morphology: Bioacoustics, genetics, and internal tadpole morphology reveal a new species of glassfrog (Anura: Centrolenidae: Ikakogi) from the Sierra Nevada de Santa Marta, Colombia
Source: PLoS One. 2019 May 8;14(5):e0215349. doi: 10.1371/journal.pone.0215349 (PMC6506205; doi:10.1371/journal.pone.0215349)
Supplement: S1 Appendix — (DOCX) [file pone.0215349.s001.docx]

Appendix S1. Tadpoles of *Ikakogi tayrona* were collected on April 11, 2015 in the municipality of Santa Marta, Magdalena Department, NE flank of SNSM, Colombia (San Lorenzo biological station, San Lorenzo stream, 11° 06’ 54.96” N, 74° 03’ 03.46” W, 2100 m elevation, and on April 18, 2016 in the headwaters of Gaira river, 11° 10' 2.0" N, 74° 10' 41.5" W, 1560 m), CBUMAG: ANF 00960 and 01017. Tadpoles of *Ikakogi ispacue* sp nov., CBUMAG: ANF 01015, and voucher specimens were recorded and/or collected on April 1, 2015 and October 1, 2016 in the municipality of Dibulla, Guajira Department, N flank of SNSM, Colombia (small tributary of Palomino River, 11°07'29.3" N; 73°33'23.5" W, 950 m elevation), CBUMAG: ANF 00938 and ICN 56201 (two individuals SVL= 33.5 and 32.6 mm, respectively).
