# Supplementary material for: The poverty of adult morphology: Bioacoustics, genetics, and internal tadpole morphology reveal a new species of glassfrog (Anura: Centrolenidae: Ikakogi) from the Sierra Nevada de Santa Marta, Colombia
Source: PLoS One. 2019 May 8;14(5):e0215349. doi: 10.1371/journal.pone.0215349 (PMC6506205; doi:10.1371/journal.pone.0215349)
Supplement: S2 Appendix — A single asterisk (*) indicates no voucher specimens collected but species indentity inferred through examination of call by the authors. Double asterisk (**) denotes examination of photographs assigned to Ikakogi but with identity uncertain. (DOCX) [file pone.0215349.s002.docx]

*Ikakogi* *tayrona* (*n =* 19): COLOMBIA: Departamento del Magdalena: Santa Marta, vereda Bella Vista, 11,080330 / -74062000, 1800 m asl, no voucher*; Santa Marta, Serranía de San Lorenzo, 4.5**–**5.5 km abajo de la estación experimental San Lorenzo, 1720-90 m asl, ICN 12997-98, 13003-4; Santa Marta, vereda La Tagua, 11,0934167 / -74,00325, 1580 m asl, no voucher*; Santa Marta, sector Norte de Ciudad Pérdida, quebrada Quiebra Patas, 11,038097 / -73,925888, 1100 m asl, ICN 8730, 12867, 12969**–**72, 12874, 12866, 12873; Santa Marta, Serranía de San Lorenzo, quebrada San Lorenzo, 11,115611 / -74,050278, 2200 m asl, no voucher*; Santa Marta, Serranía de San Lorenzo, quebrada Betoma, 11,1110 / -74,062000, 2000m asl, no voucher*; Santa Marta, Serranía de San Lorenzo, Reserva natural de la aves El Dorado, 11,108500 / -74,063389, 1950 m asl, no voucher*; Santa Marta, Serranía de San Lorenzo, alto de Mira, caño Negro, 11,038097 / -73,925888, 980 m asl, Santa Marta, Serranía de San Lorenzo, corregimiento de Minca, cascada Río Gayra, 11,111833 / -74,060889, 1560 m asl, no voucher*; Santa Marta, Serranía de San Lorenzo, corregimiento de Minca, hacienda La Victoria, 11,128944 / -74,093277, 850 m asl, no voucher; Santa Marta, Serranía de San Lorenzo, corregimiento de Minca, Pozo Azul; 11,134182 / -74,102287, 700 m asl, no voucher*; Santa Marta, Serranía de San Lorenzo, corregimiento de Minca, ca., hacienda Cincinati, quebrada Mal Abrigo, 11,08889 / -74,10947, 1500 m asl, ICN 35234; Santa Marta, corregimiento de San Pedro de la Sierra, quebrada Pascuales, 10,91944 / -73,93056, 2100 m asl, ICN 2918; Ciénaga, corregimiento Palmor, hacienda Nápoles, 10,759706 / -73,991089, 1800 m asl, no voucher*; Ciénaga, corregimiento Santa Clara, quebrada El Progreso, 10,461486 / -73,905192, 1480 m asl, no voucher; Ciénaga, Km 4. al E. de El Campano, vertiente W. del Cerro Kennedy, 11,055531 / -74,068372 , 1290 m asl, no voucher*; Ciénaga, hacienda Tierra Grata, 11,002508 / -74,092191, 1300 m asl, ICN 2903; Departamento del Cesar: Valledupar, corregimiento de Nabusimake, camino real al Atlo de Atimekeke, 10,45215556 / -73,583389, 2400 m asl, ICN 35235**–**9, 35241-3, 35245; Valledupar, Pueblo Bello, 10,415917 / -73,585167, 1200 m asl, no voucher*.

*Ikakogi ispacue* sp. nov. (*n =* 14): COLOMBIA: Departamento de la Guajira: Dibulla, corregimiento of Palomino, arroyo en la cuenca alta tributario del Río San Salvador, 11,12480556 / -73,55647222, 950 m asl, ICN 56198**–**56210, and CBUMAG:ANF 00938 (LARS 112); Riohacha, Monte Cheturrycuak, sitio La Cueva, Resguardo Wiwa, cuenca alta del Río Tapias, 11,058091/ -73.227770, 850 m asl, RC.

*Ikakogi* sp (*n =* 1): Departamento de Guajira, Mamarongo, cabecera del Río Rancheria, 10.948613 / -73.324710, 2200 m asl, no voucher** (uncertain identity).
