# Supplementary material for: The poverty of adult morphology: Bioacoustics, genetics, and internal tadpole morphology reveal a new species of glassfrog (Anura: Centrolenidae: Ikakogi) from the Sierra Nevada de Santa Marta, Colombia
Source: PLoS One. 2019 May 8;14(5):e0215349. doi: 10.1371/journal.pone.0215349 (PMC6506205; doi:10.1371/journal.pone.0215349)
Supplement: S3 Appendix — (DOCX) [file pone.0215349.s003.docx]

Appendix S3. Realigned Alignment of 2 sequences: *Ikakogi tayrona* (MAR_545) and *Ikakogi ispacue* sp. nov. (ICN 56200)

Identities = 316/371 (85.2%), Positives = 316/371 (85.2%), Gaps = 0/371 (0%).

| Species | Pb | Sequence | Pb |
| --- | --- | --- | --- |
| *I. tayrona* | 1 | TAAAAATTATTAATAACTCATTTATTGACCTACCAGCACCAGCTAACCTGTCATCATGAT | 60 |
|  |  | TAAAAATT  T  AA AACTCATTTATTGAC  TACCAGCACCAGCTAACCT  TCATCATGAT | |
| *I. ispacue* | 1 | TAAAAATTGTCAACAACTCATTTATTGACTTACCAGCACCAGCTAACCTATCATCATGAT | 60 |
|  |  |  |  |
| *I. tayrona* | 61 | GAAACTTTGGATCCCTTCTGGGGGTATGCTTAATTGCACAAATTGTAACAGGATTATTTC | 120 |
|  |  | GAAACTTTGG  TC  CT  CT GGGTATGC  TA  TTGCACAAATTGT  ACAGGA  TATT  C | |
| *I. ispacue* | 61 | GAAACTTTGGCTCTCTACTAGGAGTATGCCTAGTTGCACAAATTGTCACAGGACTATTCC | 120 |
|  |  |  |  |
| *I. tayrona* | 121 | TAGCCATACACTACACGGCAGACACTACAATAGCTTTTTCATCAATTGCCCACATTTGCC | 180 |
|  |  | TAGCCAT  CACTA  AC  GCAGA  AC  ACAAT  GC  TTTTCATCAAT  GCCCA  ATTTG C | |
| *I. ispacue* | 121 | TAGCCATGCACTATACTGCAGATACCACAATGGCCTTTTCATCAATCGCCCATATTTGTC | 180 |
|  |  |  |  |
| *I. tayrona* | 181 | GAGACGTTAACAACGGATGGCTCCTACGAAACCTTCATGCAAACGGAGCTTCATTTTTCT | 240 |
|  |  | G  GACGTTAACAA  GGATG  CTCCT  CGAAACCTTCA  GCAAACGGAGC  TCATTTTTCT | |
| *I. ispacue* | 181 | GGGACGTTAACAATGGATGACTCCTGCGAAACCTTCACGCAAACGGAGCCTCATTTTTCT | 240 |
|  |  |  |  |
| *I. tayrona* | 241 | TTATTTGCATCTACTTACACATTGGACGAGGAATCTACTACGGATCATTCCTCTTCAAAG | 300 |
|  |  | T  AT  TGCATCTAC  T  CACATTGGACG  GGAAT  TA  TA  GG  TCATT  CTCTT  AAAG | |
| *I. ispacue* | 241 | TCATCTGCATCTACCTGCACATTGGACGGGGAATTTATTATGGCTCATTTCTCTTTAAAG | 300 |
|  |  |  |  |
| *I. tayrona* | 301 | AAACATGAAATATTGGAGTAATTCTTCTTTTCCTAGTCATAGCTACAGCATTTGTCGGCT | 360 |
|  |  | A  ACATGAAA  ATTGG  GTAATTCT   TTT  CTAGT  ATAGC  ACAGCATT  GTCGG  T | |
| *I. ispacue* | 301 | AGACATGAAACATTGGGGTAATTCTCTTATTTCTAGTTATAGCCACAGCATTCGTCGGAT | 360 |
|  |  |  |  |
| *I. tayrona* | 371 | ACGTCCTTCCA | 371 |
|  |  | A  GTCCT  CCA |  |
| *I. ispacue* | 371 | ATGTCCTCCCA | 371 |
