# Supplementary material for: The poverty of adult morphology: Bioacoustics, genetics, and internal tadpole morphology reveal a new species of glassfrog (Anura: Centrolenidae: Ikakogi) from the Sierra Nevada de Santa Marta, Colombia
Source: PLoS One. 2019 May 8;14(5):e0215349. doi: 10.1371/journal.pone.0215349 (PMC6506205; doi:10.1371/journal.pone.0215349)
Supplement: S4 Appendix — (DOCX) [file pone.0215349.s004.docx]

| **Species** | **Voucher** | **GB accession numbers** |
| --- | --- | --- |
| *Ikakogi tayrona* | MAR545 | MK809523 |
| *Ikakogi ispacue* **sp. nov.** | ICN 56200 | MK809522 |
