# Supplementary material for: The poverty of adult morphology: Bioacoustics, genetics, and internal tadpole morphology reveal a new species of glassfrog (Anura: Centrolenidae: Ikakogi) from the Sierra Nevada de Santa Marta, Colombia
Source: PLoS One. 2019 May 8;14(5):e0215349. doi: 10.1371/journal.pone.0215349 (PMC6506205; doi:10.1371/journal.pone.0215349)
Supplement: S5 Appendix — (DOCX) [file pone.0215349.s005.docx]

Appendix S5. Cranial muscles codified for *Ikakogi tayrona* and *I. ispacue* sp. nov.

| **Muscle** | **Origin** | **Insertion** | **Comments** |
| --- | --- | --- | --- |
| **Mandibular group, n. trigeminus (c.n. V) innerved** | | | |
| *Levator mandibulae longus superficialis* | Dorsal curvature of palatoquadrate | Dorsomedial Meckel`s cartilage | Via long tendon |
| *Levator mandibulae longus profundus* | Curvature of palatoquadrate and ventral processus ascendens | External margin of the suprarostral ala | Via a long tendon; tissue mass near the upper lip |
| *Levator mandibulae externus superficialis* | *Inner processus muscularis* (superior) | Dorsal suprarostral ala | Extremely reduced |
| *Levator mandibulae externus profundus* | *Inner processus muscularis* (medial) | Suprarostral alae | Share a tendon with LMLP |
| *Levator mandibulae articularis* | *Inner processus muscularis* (inferior) | Dorsal Meckel`s cartilage |  |
| *Levator mandibulae laterais* | – | – | Absent or just a simple fiber not functional |
| *Submentalis (intermandibularis anterior)* | – | – | – |
| *Intermandibularis* | Median aponeurosis | Ventromedial Meckel`s cartilage | – |
| *Mandibulolabialis* | Ventromedial Meckel`s cartilage | Lower lip | – |
| *Levator mandibulae internus* | Ventral processus ascendes and few fibers on the lateral curvature | Distal Meckel | – |
| **Hyoid group, n. facialis (c.n. VII)** | | | |
| *Hyoangularis* | Dorsal ceratohyal | Retroarticular process of Meckel`s cartilage | Two slips inserting on Meckel; one via tendon |
| *Quadratoangularis* | Ventral palatoquadrado | Retroarticular process of Meckel`s cartilage | – |
| *Suspensorioangularis* | Ventral palatoquadrado and processus muscular | Retroarticular process of Meckel`s cartilage | Very large |
| *Orbitohyoideus* | *Processus muscularis* | Lateral edge of ceratohyal | There is fiber tissue between the PM and PAO; some fibers on it |
| *Suspensoriohyoideus* | Posterior descending margin of *processus muscularis* and arco subocular | Lateral process of ceratohyal | Most straight, with some fibers backwards |
| *Interhyoideus* | Median aponeurosis | Ventral ceratohyal |  |
| **Branchial group, n. Glossopharyngeus (c.n. IX) and vagus (c.n. X)** | | | |
| *Levator arcus branchialium I* | Arco subocular | Ceratobranchial I | Singlepack of fibers |
| *Levator arcus branchialium II* | Lateral otic capsule | Ceratobranchial II | – |
| *Levator arcus branchialium III* | Dorsal otic capsule | Ceratobranchial III | – |
| *Levator arcus branchialium IV* | Ventral otic capsule | Ceratobranchial IV | – |
| *Tympanopharyngeus* | Otic capsule | Ceratobranchial IV and *pericardium* | – |
| *Dilator laryngis* | Otic capsule | Arytenoid cartilage | – |
| *Constrictor branchialis I* | - | – |  |
| *Constrictor branchialis II* | *Processus branchialis* II | *Commissura terminalis* I | By the inner margin of CB I |
| *Constrictor branchialis III* | *Processus branchialis* III | *Commissura terminalis* II | By the inner margin of CB II |
| *Constrictor branchialis IV* | Ceratobranchial III | *Commissura terminalis* II | Along the CB III |
| *Subarcualis rectus I* | Posterior lateral base of ceratohyal | *Processus branchialis* II and III and ceratobranchial I | – |
| *Subarcualis rectus II-IV* | Ceratobranchial IV | *Processus branchialis* II | Crossing the CB III; inserted as well in the PB III |
| *Subarcualis obliquus II* | *Processus urobranchialis* | *Processus branchialis* II and III | – |
| *Diaphragmatobranchialis* | Peritoneum (diaphragm) | Distal Ceratobranchial III | – |
| **Spinal group, spinal nerve innervation** | | | |
| *Geniohyoideus* | Hypobranchial plate | Infrarostral cartilage | Between CB II and III |
| *Rectus abdominis* | Peritoneum (diaphragm) | Abdominal wall | Six open myomers; sixth is the largest |
| *Rectus cervices* | Peritoneum (diaphragm) | Processus branchialis III |  |
| *Interhyodeus posterior* | Present, continuous | – | – |
| *Diaphragmatoparaechordalis* | Present, arch | – | – |
